# Biological and Clinical Applications of Dimethyl Sulfoxide: Evidence and Safety Considerations

**DOI:** 10.3390/biom16101534

**Published:** 2026-10-21

**Authors:** Zihan Huang, Jia Wang

**Affiliations:** School of Health Science and Engineering, University of Shanghai for Science and Technology, Shanghai 200093, China

**Keywords:** dimethyl sulfoxide, interstitial cystitis/bladder pain syndrome, musculoskeletal pain, central nervous system injury, cardiovascular injury, cryopreservation

## Abstract

Dimethyl sulfoxide (DMSO) is a polar aprotic solvent used in biomedical research, pharmaceutical formulation, some clinical applications, and cryopreservation. Here we review molecular activity related to DMSO and selected clinical applications including interstitial cystitis/bladder pain syndrome (IC/BPS), musculoskeletal and traumatic injury, central nervous system (CNS) trauma, cardiovascular and ischemic injury, historical oncology research, local applications, cryopreservation and safety. Of the applications discussed, intravesical DMSO has the clearest contemporary clinical support in selected patients with IC/BPS, particularly those with Hunner-type disease, although its role in non-Hunner disease remains uncertain. This concentration may partly reflect DMSO’s limited regulatory approval for therapeutic use, rather than comparative evaluation across indications. Mixed findings have been reported for other applications: modest pain relief has been observed in knee osteoarthritis, whereas no benefit has been found in systemic sclerosis ulcers, where substantial skin intolerance has also been reported. Small uncontrolled clinical series reported reductions in intracranial pressure following intravenous DMSO administration. Walker’s monograph separately described neurological recovery after CNS trauma, but concurrent treatment and incomplete documentation limit causal attribution. Reduced intracranial pressure alone does not establish long-term neurological benefit. Historical oncology reports described DMSO-containing hematoxylin preparations, including “haematoxylon”, but evidence of antitumor efficacy remains insufficient. Historical ocular and otic applications involved diverse formulations but limited controlled clinical assessment. Cardiovascular protection remains supported mainly by preclinical research. DMSO is an effective cryoprotectant but residual exposure may cause infusion reactions and removal after thaw adds to the processing burden and potential cell loss. Biological activity and risk vary with concentration, route, duration, formulation, material quality, and biological setting. Historical narratives identify questions for further study but do not establish clinical efficacy. This review summarizes reported DMSO effects and risks, examines study limitations, and identifies unresolved research questions.

## 1. Introduction

Dimethyl sulfoxide (DMSO), as a solvent, cryoprotectant, and permeability enhancer, has been widely applied in biological and clinical research and is characterized chemically as an amphiphilic organosulfur compound [1]. Its physicochemical properties enable broad utility but also introduce interpretive challenges. Researchers widely use DMSO as an experimental vehicle, yet DMSO also produces measurable biological effects that may alter experimental outcomes. These effects do not establish therapeutic efficacy. Researchers should therefore distinguish technical, mechanistic, and clinical interpretations across application domains.

DMSO use spans multiple biomedical contexts with distinct evidentiary structures. In interstitial cystitis/bladder pain syndrome (IC/BPS), intravesical administration is the most clinically established application, though effects vary with patient phenotype, instillation regimen, and outcome definition [2]. Topical and ocular applications are primarily formulation-dependent and remain limited by heterogeneous clinical validation [3]. In cell therapy and cryopreservation, DMSO functions as a process reagent, defined by post-thaw viability, functional recovery, and product integrity [4,5]. These distinct uses should therefore be evaluated according to route, purpose, and outcome rather than treated as a single intervention [5].

A key challenge in interpreting DMSO-related research is the tendency to conflate solvent-associated biological activity observed in experimental systems with therapeutic effects in clinical settings. Controlled experiments show that DMSO alters membrane organization, protein environment, and cellular signaling under defined conditions [6]. However, these mechanistic effects do not directly translate into clinical benefits. Experimental outcomes are influenced by cell type, DMSO concentration, exposure duration, and assay conditions [7]. Therefore, safety and efficacy signals should be interpreted separately across laboratory, clinical, and product handling settings. Each domain operates under fundamentally different exposure constraints and outcome metrics [8].

Early clinical development of DMSO was interrupted when clinical trials in the United States were suspended in November 1965 following reports of lens changes in experimental animals [9,10,11]. Walker later acknowledged the anecdotal nature of much of his material and described physicians who declined to be named in connection with DMSO [11]. Several early therapeutic proposals are preserved in older reports and monographs, whereas the extent of unpublished research remains undetermined from these sources. This fragmented record presents a review challenge: excluding historical narratives may omit informative research directions, whereas treating them as verified clinical findings would overstate efficacy. We therefore discuss such accounts separately from controlled studies and explicitly identify their methodological limitations.

This review examines DMSO’s interactions with membranes and proteins, its effects on cellular stress responses, and its roles in high-throughput screening and cryopreservation. We cover clinical and experimental studies of IC/BPS, musculoskeletal and traumatic injury, central nervous system (CNS) and spinal cord injury, and cardiovascular and ischemic injury. Other topics include historical investigations in oncology, drug delivery, topical, ocular, and otic applications, extravasation management, and safety by route of administration. We include unapproved therapeutic uses to bring together dispersed clinical observations and identify questions that warrant further investigation, not to endorse their clinical efficacy. Throughout the review, we compare clinical findings with historical therapeutic investigations and evidence from animal and in vitro models, with particular attention to quantitative outcomes, exposure conditions, and study limitations. This review supports cautious interpretation of DMSO-related findings and may help define priorities for future research.

### Literature Search and Article Selection

PubMed and Web of Science Core Collection were searched for publications dated from 1 January 1960 through 12 October 2026. ClinicalTrials.gov was searched on 13 October 2026 for relevant registry records. The search strategy combined DMSO-related terms with terms covering five thematic areas: IC/BPS and intravesical treatment; other clinical and historical applications; cryopreservation and cell-product handling; safety and adverse effects; and molecular mechanisms, solvent effects, and assay interference. An additional IC/BPS sensitivity search combined disease-related terms with efficacy- and safety-related terms. The thematic coverage was broadened during revision in response to reviewer and editorial comments, with additional topics selected for their relevance to historical and clinical DMSO use. The complete search strings for each database are provided in Supplementary Table S4.

Sources were eligible if they addressed DMSO as a biological exposure or as a component of a clinical or experimental preparation and provided information relevant to the topics covered in this review. Eligible sources included clinical trials, guidelines, systematic and narrative reviews, observational studies, case reports, animal studies, and in vitro investigations. No minimum sample size or study-design threshold was applied because the review included both contemporary clinical research and historical therapeutic reports. Combination-treatment studies were retained when DMSO was one of the treatment components; their outcomes were attributed to DMSO alone only when the study design permitted such attribution.

Historical monographs, archival reports, and unpublished accounts were considered when their sources were identifiable and they described a specific DMSO-related application or case. These materials were classified as historical or anecdotal and were not treated as independently verified clinical findings. Walker’s monograph and its reference list were examined for early case narratives and therapeutic proposals [11]. For historical materials summarized in Supplementary Table S2, source type, access status, and verification limitations are reported alongside each account. Verification status was classified according to the material available: original full text, abstract, bibliographic record, or secondary account.

Sources were excluded from the substantive synthesis if DMSO’s role could not be determined, the topic was outside the review’s subject areas, or the available information was insufficient to describe the source reliably. Non-English studies were considered when an English abstract or accessible original text provided enough information to assess the study design, DMSO exposure, and relevant findings. Records available only as a title, citation, or incomplete bibliographic entry were not used to support detailed quantitative or clinical claims.

The three database searches retrieved 4052 records from PubMed, 3992 from Web of Science Core Collection, and 27 from ClinicalTrials.gov, for a total of 8071 records before deduplication. Deduplication by DOI, PMID, NCT number, and bibliographic matching, followed by manual review of near-duplicate title pairs, removed 1728 records and left 6343 unique records. A complete contemporaneous log of title and abstract decisions and reasons for exclusion was not available; the screening flow therefore could not be reconstructed as an independently duplicated, prospectively documented process. We found 102 of the 122 sources cited in the final narrative synthesis among the deduplicated records retrieved from PubMed, Web of Science Core Collection, and ClinicalTrials.gov. We classified the other 20 cited sources as supplementary for reporting because they were absent from the exported records. They included methodological references, guidance or regulatory material, historical or archival sources and relevant publications not captured by the fixed database searches. We did not always record the identification of each supplementary source. Accordingly, the figure of 102 represents cited sources identified in the database search records; it does not represent a prospectively logged screening-inclusion count.

Z.H. assessed the records selected for detailed review and extracted study information. J.W. checked the extracted information against the available source materials. Record assessment and data extraction were not independently duplicated. Where available, the extracted variables included sample size, DMSO concentration and dose or volume, administration route, treatment schedule, comparator, numerical outcomes, and adverse events. For intravesical IC/BPS studies, we also recorded validated symptom measures, the Global Response Assessment, and treatment-related adverse events.

The principal studies were assessed using methods appropriate to their designs: RoB 2 for randomized trials [12], ROBINS-I 2016 for non-randomized comparative studies [13], and SYRCLE’s risk-of-bias tool for animal intervention studies [14]. Other study types were assessed descriptively according to their design and reporting limitations. Z.H. conducted the assessments using the source materials listed in Supplementary Table S3. J.W. checked the consistency of the domain-level judgments, overall judgments, and supporting rationales. Risk-of-bias appraisal was not independently duplicated.

## 2. Molecular Basis of DMSO Activity

### 2.1. Membrane and Protein-Level Interactions

In model membranes, DMSO changes lipid packing, hydration, and permeability in a concentration-dependent manner [1,15,16]. Its effects vary with hydration state, while microfluidic measurements show that transport also depends on cell type and trapping conditions [1,6]. Liposome and bilayer studies link DMSO permeation and pore formation to interfacial water behavior [15,16]. These models clarify membrane interactions but cannot predict responses in diseased human tissues.

DMSO also affects protein structure and binding environments [17]. Nerve growth factor studies illustrate its potential to alter receptor interactions, while screening assays may require higher DMSO concentrations to dissolve test compounds [17,18]. Changes in protein stability, ligand-binding kinetics, and membrane access can then influence measured activity [1,6,16,19,20]. DMSO may also affect compound solubility, precipitation, or aggregation, changing the free-compound concentration across wells [18].

These effects can produce assay drift or misleading activity calls. DMSO concentration should be reported and matched across controls, standards, and test samples. Where targets or readouts are solvent-sensitive, results should be checked with an independent assay. Findings from these simplified systems support mechanistic questions but do not establish clinical benefit.

### 2.2. Inflammatory and Cellular Stress Responses

DMSO alters inflammatory mediators, transcription, and epigenetic signals in experimental synoviocyte, hepatocyte, and human blood-cell models [7,21,22]. Solvent composition also affects protein-aggregation assays: low-dose DMSO has been linked to accelerated alpha-synuclein aggregation, while amyloid-beta results vary with vehicle matching [23,24]. DMSO-based differentiation protocols may change mitochondrial activity and inflammatory state, and bladder smooth-muscle studies suggest effects involving acetylcholinesterase [25,26].

**Figure 1:**
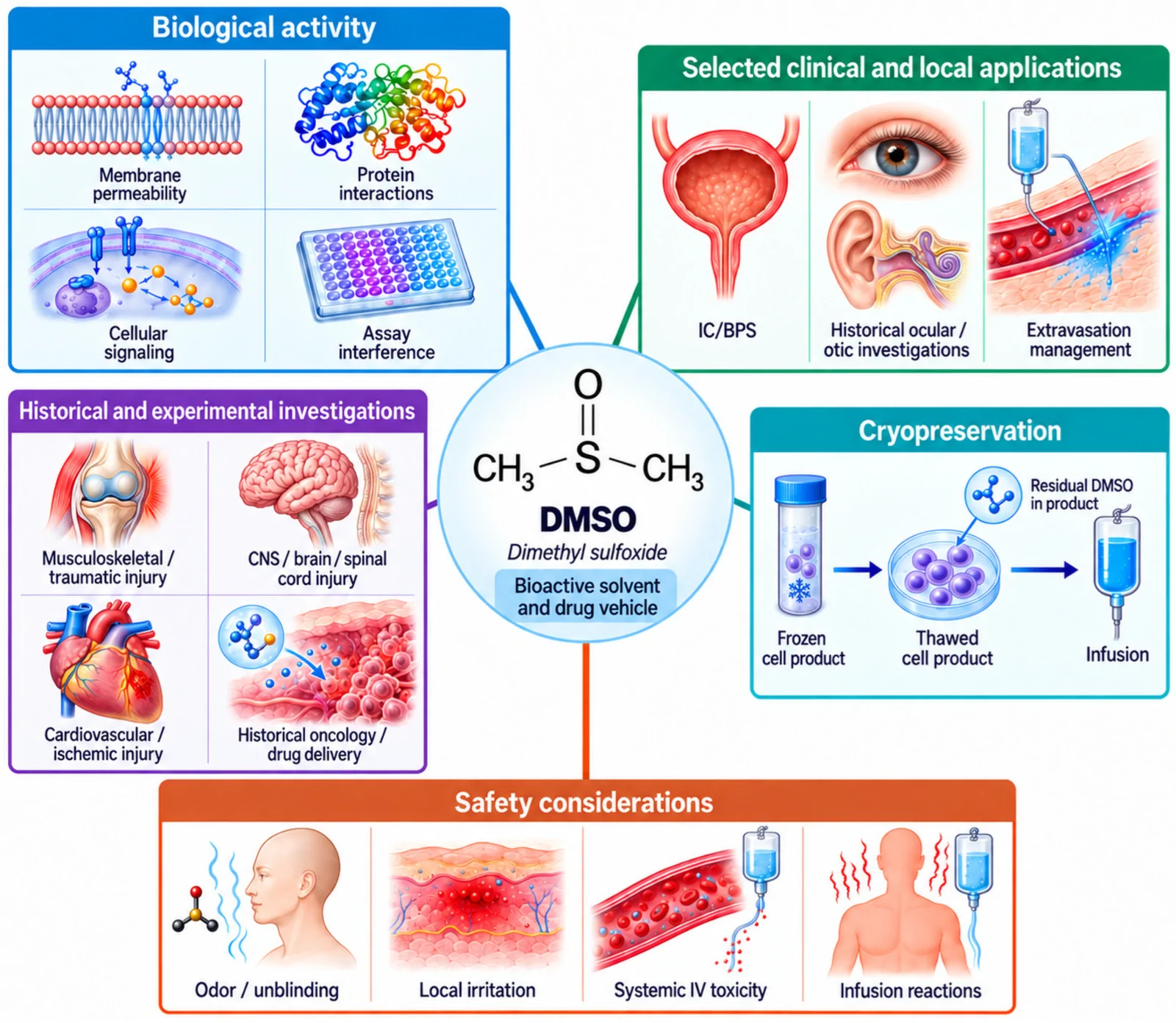
Biological activities, selected clinical and local applications, historical and experimental investigations, cryopreservation, and route-specific safety considerations of DMSO. The oncology panel presents a historical proposal for drug delivery. The ocular and otic panel summarizes early empirical investigations. Neither establishes standardized clinical efficacy. CNS, central nervous system; DMSO, dimethyl sulfoxide; IC/BPS, interstitial cystitis/bladder pain syndrome.

These responses depend on concentration, exposure, and model conditions [21]. DMSO should therefore be controlled as an active experimental variable, particularly in assays of signaling, aggregation, or cytotoxicity. Such findings guide study design but do not establish therapeutic benefit [24]. Figure 1 summarizes these biological activities.

## 3. Clinical Findings in Interstitial Cystitis/Bladder Pain Syndrome

### 3.1. Clinical Positioning and Clinical Use

DMSO serves as an intravesical option for IC/BPS, mainly in selected cases. The 2022 American Urological Association guidelines list DMSO among bladder-directed therapies. These guidelines emphasize individualized symptom-based management [2]. Recent consensus statements describe intravesical therapies as part of multimodal rather than monotherapy regimens [27]. Earlier reviews also placed DMSO within a stepwise treatment context, not as universal first-line care [28].

In practice, DMSO use depends on patient characteristics, prior treatments, instillation protocols, and outcome measures [2]. Its presence in clinical documents reflects clinical experience rather than defined molecular mechanisms [27]. Therefore, DMSO is not used uniformly across all bladder pain patients. It mainly provides symptom control in selected individuals [28]. Reviews of intravesical IC/BPS therapy emphasize that treatment selection should consider symptom burden, prior failure, and patient tolerance [28].

### 3.2. Phenotype-Related Treatment Response

A Japanese multicenter, double-blind phase III trial randomized 96 patients to intravesical 50% DMSO (n = 49) or placebo (n = 47), administered every two weeks for 12 weeks [29]. All 96 patients comprised the full analysis set, whereas week-12 ICSI measurements were available for 48 and 45 patients, respectively. The ICSI ranges from 0 to 20, with higher scores indicating greater symptom severity. The least-squares mean changes from baseline were −5.2 and −3.4 points, with a between-group difference of −1.8 points (95% CI, −3.3 to −0.3; *p* = 0.0188). Week-12 Global Response Assessment (GRA) responder rates were 25/48 (52.1%) and 14/46 (30.4%), respectively (*p* = 0.0385) [29].

A post hoc analysis included 83 patients with Hunner-type IC, representing 86.5% of the full analysis set (42 receiving DMSO and 41 receiving placebo) [30]. Among patients with available week-12 GRA data, 22/41 (53.7%) and 11/40 (27.5%) were responders, respectively (*p* = 0.0236). The non-Hunner subgroup included only 13 patients. This small sample leaves the treatment effect in non-Hunner disease uncertain and does not establish a difference in response between phenotypes [30].

These findings support short-term symptom improvement in the studied predominantly Hunner-type population, but confirmation is needed because treatment regimens, patient selection, comparators, and symptom measures vary across studies [28,30,31]. Pooled improvements should therefore not be taken as a uniform effect across IC/BPS populations [28].

### 3.3. Variability Across Studies and Treatment Regimens

Intravesical DMSO regimens vary in composition and schedule. Stav et al. treated 51 patients weekly for 12 weeks with a mixture containing 50 mL of a 50% DMSO solution, hydrocortisone (100 mg in 5 mL), heparin sodium (10,000 U in 10 mL), and 10 mL of 0.5% bupivacaine [32]. The listed component volumes totalled 75 mL. The reported 50% concentration referred to the DMSO solution added to the mixture, rather than the final preparation. Moss et al., in a prospective randomized study, used 50 mL of DMSO and 1 mL of triamcinolone (10 mg/mL), administered weekly for six weeks. Each dose had a total volume of 51 mL. The available full text did not specify the DMSO stock concentration or its concentration basis; therefore, the final DMSO concentration could not be determined from the reported information. The comparator formulation comprised 30 mL of 0.5% bupivacaine, 2 mL of triamcinolone (10 mg/mL), and 2 mL of heparin (10,000 U/mL) [33].

Direct comparison is limited by differences in volume, co-administered drugs, and treatment duration [32,33]. Symptom improvement in combination regimens cannot be attributed to DMSO alone because co-administered agents may have independent effects [33,34,35]. Studies should report patient characteristics, formulation, schedule, and outcome definitions, and distinguish DMSO monotherapy from combination or post-procedural use where possible [28,33].

### 3.4. Interpretation of Controlled and Randomized Studies

Study design affects the attribution of benefit to DMSO. In multi-agent instillations containing bupivacaine, triamcinolone, or heparin, symptom changes cannot be assigned to DMSO alone [33]. By contrast, the Japanese multicenter, double-blind, placebo-controlled phase III trial permitted clearer attribution of symptom improvement to DMSO [29]. Combination studies may better reflect routine clinical practice, whereas single-agent trials provide more direct treatment-specific comparisons. Both types of studies remain subject to differences in patient selection, treatment regimen, baseline severity, dwell time, treatment frequency, concurrent medication, and outcome assessment [30,33]. These variables should be reported consistently so that short-term relief is not mistaken for sustained improvement. Validated measures, including the Interstitial Cystitis Symptom Index and Global Response Assessment, improve comparability but do not eliminate protocol-related differences [29,30].

The statistically significant ICSI difference requires consideration alongside the improvement observed with placebo. The −1.8-point between-group estimate represents an additional average reduction beyond the −3.4-point change in the placebo group [29]. In the post hoc analysis, a five-point within-patient ICSI reduction was associated with patient-reported improvement in the pooled Hunner-type population [30]. The within-patient threshold cannot be directly applied to the between-group mean difference. The trial population was predominantly Hunner-type, which limits generalization to IC/BPS populations with a different phenotype distribution [29,30]. Clinical relevance should be considered alongside GRA response, tolerability, and durability of improvement.

A 2026 ex vivo study of pig and human bladder tissue found concentration-dependent but reversible inhibition of detrusor and mucosal contractility, followed by enhanced mucosal contractile activity after washout. This observation may help frame transient post-instillation symptoms, but it does not establish clinical efficacy [36].

### 3.5. Research Needs and Clinical Perspective

Future IC/BPS studies should classify Hunner and non-Hunner disease separately and report baseline severity, DMSO concentration, dwell time, treatment frequency, comparators, co-administered drugs, and follow-up [29,31]. Safety, short-term symptom relief, and sustained response should be assessed as distinct outcomes [28]. DMSO remains one bladder-directed option within broader care, but inconsistent patient grouping and reporting limit comparisons across studies [37,38].

Experimental models can inform hypotheses about inflammation and epithelial changes, particularly in Hunner-type disease, but cannot establish clinical benefit [39]. The central question is which patients achieve lasting improvement under defined treatment conditions [29]. Principal intravesical studies and their quantitative findings are summarized in Table 1.

## 4. Musculoskeletal Pain, Soft-Tissue Conditions, and Traumatic Injury

Early reports explored DMSO for rheumatoid and other musculoskeletal conditions but did not establish a consistent treatment effect [47,48,49]. Later controlled trials produced mixed findings. Eberhardt et al. randomized 112 patients with active knee osteoarthritis to 25% DMSO gel or placebo for three weeks. The primary abstract reported less pain with DMSO during loading (*p* = 0.019), at rest (*p* = 0.015), and on palpation (*p* = 0.029), without serious adverse events [50]. A later secondary extraction estimated an 11.7 mm greater improvement in loading pain with DMSO (95% CI, 5.1–18.35) [50,51].

In a separate trial, Williams et al. assigned 84 patients with systemic sclerosis and digital ulcers to topical 2% DMSO, 70% DMSO, or saline. Ulcer and pain outcomes did not differ significantly, while more than one-quarter of the 70% DMSO group withdrew because of skin toxicity. The available report did not provide group-specific sample sizes or the exact withdrawal count [52]. These trials do not support a broad therapeutic claim across the conditions studied. Representative studies outside IC/BPS are summarized in Table 2.

## 5. Central Nervous System, Brain, and Spinal Cord Injury

Clinical series with numerical data focused on intracranial pressure (ICP). Marshall et al. treated six patients intravenously: two received rapid boluses of 10% DMSO, and four received 20% DMSO titrated to ICP. ICP was initially controlled, but all six developed fluid or electrolyte problems, and DMSO damaged standard infusion systems [57]. Karaca et al. treated 10 patients with severe head trauma using 28% DMSO (1.12 g/kg every 6 h for 1–10 days); four also received intermittent oxygen. Mean ICP reductions at 24 h were 28 mmHg with DMSO alone and 39 mmHg with added oxygen. Seven patients reached normal ICP by day 6, and two died [58]. The uncontrolled design limits causal attribution, and the reported ICP reductions do not establish a long-term neurological benefit attributable to DMSO.

Early experimental and review literature also discussed the timing of DMSO administration in CNS injury [59,60]. Preclinical findings vary by model. After spinal-cord injury, partial or complete recovery occurred in 6/8 DMSO-treated dogs versus 2/8 given dexamethasone; the DMSO dose was not reported [61]. Rhesus-monkey brain-injury work reported physiological changes without reported group sizes or numerical effect estimates [62]. DMSO reduced CA1 neuronal loss in a gerbil ischemia model, while a rat traumatic-brain-injury study reported behavioral and oxidative-stress changes [63,64]. These studies are detailed in Supplementary Table S1. Together, they suggest questions for further study, but lasting neurological benefit has not been demonstrated in controlled human studies.

Walker separately described neurological recovery after intravenous DMSO in patients with severe spinal injuries, including a reported case following cervical trauma [11]. These accounts are summarized in Supplementary Table S2. The original clinical records and patient-level treatment details were not independently verified, and concurrent intensive care and rehabilitation prevent attribution of the reported recovery to DMSO. These reports are regarded as historical narratives rather than evidence that clinical efficacy has been established.

## 6. Cardiovascular and Ischemic Injury

Interest in DMSO for cardiac and ischemic injury dates to early myocardial-necrosis studies [60,65,66], but support for cardioprotection remains largely preclinical. In rats given 500 μL/kg intraperitoneally for three days before coronary ligation, Parisi et al. reported lower myocardial damage (4.46 ± 2.01% vs. 18.75 ± 4.88%), troponin I (2.95 ± 1.32 vs. 29.35 ± 12.32 ng/mL), and myoglobin (13.75 ± 0.85 vs. 46.86 ± 10.35 ng/mL) than in untreated controls (all *p* < 0.05). Group sizes varied by assay, and pretreatment limits clinical relevance [67]. Dmitriev et al. also reported reduced infarct size after intraperitoneal pretreatment and less postischemic ventricular dysfunction with intraperitoneal or intracoronary DMSO, but dose, group sizes, and numerical effect estimates were NR [68].

Systemic exposure raises separate safety concerns. In six dogs, intravenous DMSO (2 g/kg) caused transient hemodynamic changes that largely moved toward baseline within 10 min [69]. Among 22 recipients of DMSO-containing grafts, seven asymptomatic sinus-bradycardia episodes occurred; this report assessed infusion safety, not cardioprotection [70]. These findings do not establish cardiovascular benefit in humans. Further animal and model studies are summarized in Supplementary Table S1.

## 7. Historical Investigations of DMSO in Oncology and Drug Delivery

Historical oncology investigations examined DMSO primarily as a solvent and carrier for poorly soluble compounds. Tucker and Carrizo reported a DMSO-containing preparation for recurrent neoplasms [71]. The original report termed the material “haematoxylon”; its description as a crystalline substance with the molecular formula C_16_H_14_O_6_ supports its identification as hematoxylin. Walker later summarized a 37-patient series involving advanced malignancies [11]. Walker also cited a related unpublished manuscript by Rogers from 1979; this manuscript was not independently obtained or examined [11]. Rogers’s 1970 doctoral dissertation examined a DMSO–hematoxylin combination in a transplantable lymphosarcoma model [72]. These sources differ substantially in type and verifiability and should not be interpreted as a single clinical evidence set.

These reports involved heterogeneous cancer types, concurrent treatments, incomplete clinical documentation, and no controlled comparison isolating the contribution of DMSO. The published Tucker and Carrizo report is indexed without an abstract, and the relationship between Rogers’s archival dissertation and the unpublished 1979 manuscript could not be independently established. A contemporaneous oncology assessment classified the combined haematoxylon–DMSO approach as unproven [73]. Reported tumor regression or clinical improvement therefore cannot be interpreted as confirmation of an independent antineoplastic effect of DMSO.

These historical materials are included because an early drug-delivery hypothesis and an overlooked therapeutic research direction are documented in them. The proposed use of DMSO to improve the solubility and delivery of a co-administered compound is described, but clinical efficacy is not established. The available reports should therefore be distinguished from controlled oncology studies and interpreted as historical accounts requiring independent verification.

## 8. Topical, Ocular, Otic, and Extravasation-Related Clinical Uses

### 8.1. Topical, Ocular, and Otic Applications

Topical DMSO has been studied in condition-specific formulations, including preparations for pain or pruritus, but these reports do not establish efficacy across skin disorders [74,75]. Historical ocular and otic applications of DMSO were also reported, although the formulations and treatment protocols were not standardized. Walker described an ophthalmic preparation containing 5 mg of DMSO in 1 cc of normal saline, corresponding to approximately 0.5% *w*/*v*, for retrobulbar administration. Reported applications included corneal swelling and other ocular conditions [11]. In a separate report involving 50 patients with retinal degeneration, improvements in visual acuity, visual fields, and dark adaptation were described in 22, 9, and 5 patients, respectively (DMSO concentration not reported) [76]. For otic use, a historical report involving 15 patients with chronic tinnitus described complete symptom disappearance in 9 patients, substantial improvement in 2, and altered but persistent symptoms in 4 patients after treatment with DMSO-containing preparations. The external-ear treatment included 2 mL of a DMSO-containing spray (DMSO concentration not reported) [11]. These historical, uncontrolled reports do not establish standardized ophthalmic or otic efficacy.

Concentration may affect ocular responses. In an experimental inflammation model, 30% DMSO showed anti-inflammatory activity, whereas 90–100% DMSO aggravated inflammation; 0.1% dexamethasone was more effective than 30% DMSO [77]. These findings do not establish the safety or efficacy of ophthalmic DMSO in patients. Poliakova and colleagues reported the use of various drugs combined with a dimethyl sulfoxide solution in 49 patients with external-ear inflammation and 10 patients with acute otitis media [78]. The contribution of DMSO cannot be distinguished from that of the accompanying drugs. Separate assessment is required for each ocular or otic preparation, including its concentration, route, sterility, co-administered agents, and local tolerance. The available reports do not support a general claim of clinical efficacy.

### 8.2. Extravasation Management

Consensus recommendations and clinical reviews emphasize drug-specific management of antineoplastic extravasation [79,80]. Topical DMSO has a limited, drug-specific role in extravasation management. The eviQ guidance lists DMSO with local cooling for selected agents, including conventional doxorubicin, epirubicin, and mitomycin, but explicitly excludes DMSO for liposomal doxorubicin extravasation [81]. Dexrazoxane may be used as an alternative treatment for anthracycline extravasation, whereas its combination with DMSO is discouraged by the eviQ procedure because its efficacy may be reduced by DMSO [82]. Treatment selection should therefore be determined by the extravasated drug and its formulation, rather than solely by the presence of tissue injury.

Two prospective uncontrolled studies provide clinical observations. Topical 99% DMSO was administered every 6 h for 14 days by Olver et al. to 20 patients with anthracycline extravasation, with a median interval to application of 25 min. No cases of ulceration or surgical intervention were observed, although only 16 patients completed the 3-month follow-up. Burning, itching, erythema, and mild scaling were reported as adverse effects [83]. A total of 144 patients with extravasation caused by several cytotoxic drugs were enrolled by Bertelli et al., of whom 127 were assessable. Treatment was performed using topical 99% DMSO every 8 h for 7 days, accompanied by local cooling for 1 h three times daily during the first 3 days. One patient developed ulceration. Mild local burning and characteristic breath odor were reported [84].

A concurrent control group was not included in either study. In the Bertelli study, the specific contribution of DMSO cannot be isolated because local cooling was administered concurrently, while comparisons are further complicated by differences in the extravasated agent, injury extent, and treatment delay. Assessment of later tissue injury is also limited by incomplete follow-up. Consequently, the magnitude of benefit attributable to DMSO cannot be established from these observations, nor can extension of its use to all drug extravasations be justified.

### 8.3. Interpretation Across Local Applications

Case-based literature reinforces that extravasation management relies on early detection and drug-specific treatment rather than delayed or nonspecific topical approaches [85]. A pilot study using 30% DMSO cream with topical amphotericin B shows feasibility in a defined dermatologic setting [56]. However, these results cannot be directly extended to ocular conditions, pain management, or oncology-related extravasation care.

Across all local uses, factors such as formulation design, tissue characteristics, comparator treatments, and tolerability remain the main determinants of outcome interpretation [3]. A DMSO-containing topical preparation designed for one indication cannot be assumed applicable to other clinical settings without separate supporting data [74]. Therefore, delivery effects, supportive-care use, and therapeutic efficacy should be considered separately when interpreting the literature.

Overall, these studies show that the clinical role of DMSO in local applications is highly context-specific [3]. Its use is best understood within each defined clinical setting rather than as a general topical therapeutic agent [79]. A representative quantitative study of a DMSO-containing topical formulation is included in Table 2.

## 9. Cryopreservation and Cell-Product Handling

### 9.1. DMSO as a Cryoprotectant and Functional Role in Cell Preservation

DMSO enters cells and limits ice-related injury during freezing [4]. It remains an effective cryoprotectant, although reduced-DMSO and DMSO-free methods are being studied to lower residual exposure without compromising post-thaw function [86]. The appropriate DMSO concentration varies according to cell type, freezing protocol, storage duration, and intended use. Conventional hematopoietic stem cell protocols commonly use 5–10% (*v/v*) DMSO, whereas some reduced-DMSO approaches assess approximately 2.5–5% [5]. Representative studies are summarized in Table 3.

In a 2026 platelet study, platelets were cryopreserved with either 10% (*v*/*v*) DMSO or 5% DMSO after pre-freeze supernatant removal. Better preservation of in vitro platelet recovery, morphology, glycoproteins, aggregation, and clot function was observed with 10% (*v*/*v*) DMSO, although clinical superiority cannot be established from these laboratory outcomes alone [87].

DMSO is a cell-processing component, not a treatment administered for its own therapeutic effect [4]. Preservation studies should therefore assess cell recovery, viability, functional activity, product quality, and infusion tolerance together, rather than DMSO concentration alone [5,86].

**Table 3:** Quantitative studies of DMSO in cryopreservation and cell-product handling.

Study/Product	Design and Sample	DMSO Condition	Processing/Storage	Endpoints	Quantitative Findings	Scope/Source
Mitrus et al., 2018; autologous PBSC	Prospective randomized study; 150 assigned, 143 transplanted	10%, 7.5%, or 5% DMSO	Clinical cryopreservation and infusion	Infusion AEs and engraftment	Infusion AEs were lowest with 5% vs. 10% DMSO (*p* = 0.02); median leukocyte/neutrophil recovery was 10 days and platelet recovery 15 days in all groups	[88]
Gokarn et al., 2023; hematopoietic stem-cell grafts	26 graft samples (24 autologous, 2 allogeneic)	Final 4.35% DMSO with methylcellulose	Uncontrolled-rate freezing at −80 °C; median storage 6.6 years (range 3.8–11.5)	CD34+ recovery and viability	Median CD34+ recovery 80.8% (47.6–96.2); viability 84.5% by trypan blue and 80.6% by 7-AAD	[89]
Panch et al., 2019; autologous CAR-T products	158 products from six clinical trials plus three healthy-donor runs	5% DMSO + 6% pentastarch + 4% human serum albumin	Controlled-rate freezing; liquid- or vapor-phase nitrogen storage	Manufacturing, phenotype, persistence, and response	Cryopreserved starting material reduced early viability, but final expansion, transduction, phenotype, in-vivo persistence, and clinical response did not significantly differ	[90]
Liu et al., 2010; human bone-marrow MSCs	Controlled in vitro formulation study	7.5%, 5%, or 2.5% DMSO with PEG/trehalose; 10% DMSO reference	Approximately 1 °C/min controlled-rate freezing	Post-thaw viability and differentiation	7.5% DMSO + 2.5% PEG + 2% BSA yielded 82.9 ± 4.3% viability vs. 82.7 ± 3.7% with 10% DMSO; multilineage differentiation was retained	[91]

Note: Product-quality outcomes are not interchangeable with clinical efficacy. AE, adverse event; CAR-T, chimeric antigen receptor T cell; DMSO, dimethyl sulfoxide; MSC, mesenchymal stromal cell; PBSC, peripheral blood stem cell; PEG, polyethylene glycol.

### 9.2. Reduced-DMSO and DMSO-Free Approaches in Different Cell Products

DMSO-free preservation solutions for hematopoietic grafts are being developed, as infusion reactions and residual DMSO exposure remain clinical concerns [86]. Studies on immune-cell therapies show that successful translation depends on cell type, manufacturing process, quality release criteria, and post-thaw functional testing [92]. These findings suggest that reduced-DMSO or DMSO-free methods cannot be evaluated without considering the final cell product performance [93].

Across different cell types, including stem cells, immune cells, cardiomyocytes, and tissue constructs, preservation strategies vary significantly [86,92,93,94,95,96,97,98]. A reduction in DMSO is only meaningful if cells maintain survival, recovery, phenotype stability, functional activity, and therapeutic potential [95]. In contrast, complete DMSO removal must still ensure that the biological function of the product is preserved [92]. Therefore, comparisons should not be simplified to “DMSO versus no DMSO,” but should focus on whether a preservation method maintains acceptable product quality [96].

Low-DMSO preservation has been investigated in peripheral blood stem cells [96], whereas DMSO-free protocols have also been developed for induced pluripotent stem cell-derived cardiomyocytes [95]. Preservation studies in nerve tissue and trehalose-based methods further show that different biological products have different storage requirements [97,98]. Cell survival alone is not sufficient if functional properties such as differentiation ability, electrical activity, or structural integrity are lost. These results support evaluating preservation methods by cell type rather than applying a universal replacement approach [93].

DMSO-free cryopreservation has been tested in different cell types and with different freezing protocols, which limits direct comparison. In human mesenchymal stem cells, recovery comparable to that obtained with a commercial DMSO-containing cryoprotectant was achieved with one non-cell-permeable ammonium/sulfonate zwitterion [99]. In Jurkat T cells, >90% post-thaw recovery was obtained with a serum-free formulation containing 10% glycerol and 2% betaine, provided that freezing was performed at 3 °C/min [100]. DMSO exposure is avoided with these methods, while added serum is also omitted from the glycerol/betaine protocol. Functional preservation and clinical equivalence cannot be established from recovery data alone. Phenotype, functional performance, process reproducibility, scalability, and release criteria need further assessment under clinically relevant manufacturing conditions. The DMSO-free approaches discussed here have not been validated for clinical manufacturing and should therefore be regarded as experimental preservation approaches.

### 9.3. Infusion Safety and Practical Handling Considerations

Infusion reactions remain a concern in cell therapy. Their occurrence has been linked to DMSO exposure, product volume, and patient factors [8]. Reported reactions range from gastrointestinal symptoms to cardiovascular and neurological complications [101]. Dose reduction, washing, dilution, or alternative cryoprotectants may limit exposure if post-thaw cell function is preserved [5]. DMSO serves in cell processing and preservation; it is not the therapeutic component of the product [94].

The benefit of reducing DMSO must be weighed against effects on cell recovery and function [92,96]. Automated washing and dilution have been studied for this purpose [102,103,104], but require equipment, consumables, trained personnel, and process validation [105]. Additional handling may prolong processing and reduce product yield [106]. These costs and losses should be assessed alongside any reduction in infusion reactions [101].

Studies should report residual DMSO exposure, infusion safety, cell recovery and function, cell loss, processing time, and cost together [5,101]. Concentration alone is insufficient: product characteristics, infusion conditions, and patient factors also affect tolerability [5,8].

## 10. Route-Specific Safety Considerations

Quality requirements are applicable to both the DMSO starting material and the finished preparation. Pharmaceutical-quality specifications applicable to the intended product and route of administration must be met by DMSO used in therapeutic preparations. Industrial-grade DMSO must not be substituted for material meeting the applicable pharmaceutical-quality specifications [107]. In the United States, RIMSO-50 is listed by DailyMed as a sterile 50% (*w*/*w*) DMSO product for intravesical administration [108]. Worldwide commercial availability or authorization, safety, or efficacy for other indications or routes of administration is not established by this product-specific listing.

Meeting a pharmaceutical-quality specification for the starting material alone does not guarantee that the finished preparation is of adequate quality or suitable for its intended use. Manufacturers must also assess the final product’s identity, assay, impurities, excipients, container compatibility, sterility where applicable, stability, and route-specific tolerability. The formulation and intended route of administration determine the relevant requirements.

For DMSO-containing preparations, route-specific requirements are particularly important. Quality specifications for the DMSO ingredient are provided by the USP monograph [107], whereas intravesical administration is identified and intramuscular and intravenous injection are explicitly excluded by the product labeling for sterile RIMSO-50 [108]. Thus, a material meeting an ingredient-quality specification cannot automatically be regarded as suitable for direct administration by every route. Regulatory status is dependent on the specific product, formulation, route of administration, and jurisdiction. Off-label use of an approved drug does not mean that the regulator has evaluated the unapproved indication or route as safe and effective. Such use should also be distinguished from the use of an unapproved product [109].

### 10.1. Intravesical Safety

Local tolerability was generally manageable in intravesical DMSO studies, although treatment continuation was limited in some patients. In a randomized, open-label, multicenter study of women with bladder pain syndrome/interstitial cystitis, at least one adverse event was reported by 30.56% of patients receiving 50% DMSO, while treatment-related adverse events were recorded in 22.22%, compared with 1.35% in the hyaluronic acid/chondroitin sulfate group [45]. In another randomized multicenter trial, consent was withdrawn before treatment completion by 57% of patients assigned to 50% DMSO. The reported reasons were pain during or after instillation, an intolerable garlic-like odor, and lack of efficacy [46]. During repeated intravesical treatment, bladder discomfort, urinary symptoms, and odor-related intolerance should therefore be regarded as clinically relevant outcomes. Treatment completion and withdrawal rates should be included in safety assessment, rather than relying on adverse-event severity alone.

A peak DMSO concentration of 34.5 μg/mL was measured in human milk 5 h after a single lactating patient received one RIMSO-50 treatment, and the relative infant dose was estimated at 0.45%. Infant safety cannot be established from this single-participant pharmacokinetic observation [110].

### 10.2. Topical Safety

Local skin reactions are the principal effects associated with topical exposure to DMSO-containing formulations, and their frequency and severity are influenced by formulation and exposure conditions. In a long-term open-label study involving 793 patients with knee osteoarthritis, dry skin was reported in 25.3% of participants receiving a topical diclofenac formulation containing DMSO; contact dermatitis without vesicles and with vesicles was reported in 13.0% and 9.5%, respectively. Irritation, dermatitis, erythema, and local toxicity should therefore be assessed as distinct outcomes rather than grouped into one adverse-event category. Erythema or more pronounced local irritation was also documented during treatment [111]. Experimental observations further indicate that DMSO can induce concentration-dependent cutaneous inflammatory responses, with both concentration and repeated exposure influencing the type and extent of the reaction [112].

### 10.3. Systemic and Intravenous Safety

Historical intravenous use of DMSO provides a distinct safety profile from local administration. In patients treated intravenously for spinal cord injury or cerebral edema, transient hemolysis with hemoglobinuria was reported in association with DMSO exposure, although short-term treatment did not produce measurable deterioration in renal function in one clinical series [113]. Severe hemolysis has also been reported after intravenous DMSO administration [114]. Historical treatment of intracranial hypertension involved additional concerns, including fluid overload and severe electrolyte disturbances during administration of 10–20% DMSO solutions [57]. Extreme serum hyperosmolality has also been described during intravenous treatment, with one reported peak of 717 mOsm/kg [115]. Cardiovascular responses have been observed after systemic DMSO exposure [69]. The solvent properties of DMSO can also affect infusion equipment; in an early clinical series of patients treated for intracranial hypertension, mechanical difficulties occurred because DMSO progressively dissolved components of standard intravenous infusion systems [57]. These historical findings indicate that systemic exposure involves risks that are not represented by the local tolerability data obtained from intravesical or topical administration.

### 10.4. Safety of DMSO-Containing Cryopreserved Cell Products

Acute reactions have been reported during infusion of DMSO-containing cryopreserved cell products. In a single-center study of 1269 hematopoietic stem cell product infusions, adverse events occurred in 37.8%. Among the 480 infusions with adverse events, facial flushing occurred in 39.4%, nausea or vomiting in 38.1%, hypoxia requiring oxygen in 29.0%, and chest tightness in 16.7% [116]. In an earlier series of 22 recipients of products containing 10% DMSO, half developed symptoms; 7 of 8 patients who received more than 30 mL of DMSO had an infusion reaction [117]. Reported events also included blood-pressure and heart-rate changes, fever, chills, and, in a separate report, transient global amnesia [117,118]. Risk may depend on residual DMSO exposure, product volume and composition, infusion conditions, and patient characteristics; these reports do not isolate the contribution of DMSO.

### 10.5. Laboratory and Experimental Toxicity

Laboratory and model studies describe biological responses that differ fundamentally from clinical adverse events. DMSO exposure has been associated with changes in gene expression, epigenetic regulation, cellular stress responses, and developmental phenotypes in bacterial, zebrafish, and mammalian models under defined experimental conditions [119,120,121]. These findings are relevant to experimental design and interpretation, particularly because DMSO is widely used as a solvent or vehicle. However, concentrations and exposure durations used in vitro or in animal models should not be treated as equivalent to clinical exposure, and experimental molecular or developmental changes should not be converted directly into clinical adverse-event rates.

### 10.6. Summary of Safety Findings

Across applications, DMSO safety varies with route, concentration, dose or volume, exposure duration, formulation, and biological setting. Intravesical use may cause bladder discomfort, urinary symptoms, and odor-related intolerance that can lead to withdrawal. Topical formulations mainly cause local skin reactions. Hemolysis, hemoglobinuria, hyperosmolality, electrolyte disturbances, and cardiovascular effects have been associated with historical intravenous DMSO use. Acute reactions, including nausea, flushing, hypoxia, and cardiovascular or neurological events, may be caused by the infusion of cryopreserved cell products. Molecular and developmental changes observed in laboratory or animal studies should be assessed separately from clinical adverse events. Quantitative findings by administration route are summarized in Table 4.

## 11. Limitations and Future Directions

This narrative review is subject to limitations. PubMed, Web of Science Core Collection, and ClinicalTrials.gov were searched, with additional literature identified through reference-list screening and targeted citation tracing. Embase, Scopus, and the Cochrane Library were not searched, and database coverage was therefore incomplete. A complete contemporaneous screening log, including record-level exclusion decisions and reasons, was not available; consequently, the selection process could not be reconstructed as an independently duplicated, prospectively documented workflow. The exact discovery route was also not recorded consistently for all supplementary sources. Some early or non-indexed reports may therefore have been missed. Relevant non-English studies may also have been underrepresented when an accessible abstract or original text was unavailable. Historical and unpublished materials were considered when they could be identified, although limited access to original sources prevented independent verification of some accounts.

Walker’s monograph cites historical publications, including non-English literature. Additional case narratives are available in privately maintained, non-peer-reviewed online sources (e.g., https://www.midwesterndoctor.com/p/dmso-is-a-miraculous-therapy-for-657 accessed on 19 October 2026), but we did not include these accounts because their underlying records could not be independently verified. Further review of such material, together with independent verification where feasible, may help identify questions for future controlled clinical studies.

The included studies also have substantial limitations. Small samples, inadequate controls, incomplete reporting, and short follow-up limit conclusions about sustained benefit. IC/BPS studies differ in phenotype, instillation schedule, accompanying drugs, and outcome measures. DMSO’s odor and local irritation may compromise blinding. Concurrent treatment and incomplete historical records further restrict attribution of reported improvement to DMSO. Results from cell and animal models are not suitable for determining therapeutic doses or clinical benefit in humans. Cryopreservation studies differ in cell type, freezing and thawing protocols, residual DMSO exposure, and post-thaw evaluation. Cell viability alone does not guarantee functional recovery or clinical tolerability.

The structured appraisals showed that incomplete reporting, missing outcome data, subjective endpoints, and limited access to full reports materially restrict interpretation of several principal studies. Judgments based only on abstracts or bibliographic records were therefore treated as provisional rather than as confirmed absence of bias.

Future studies should incorporate indication-specific outcomes, defined exposure measures, appropriate active or vehicle controls, transparent adverse-event reporting, and follow-up long enough to distinguish transient physiological responses from lasting clinical benefit. In IC/BPS research, Hunner and non-Hunner disease should be analyzed separately, and DMSO monotherapy should be distinguished from multi-agent instillations. Studies of cryopreserved cell products should assess residual exposure, infusion safety, cell recovery, functional potency, and processing burden together. Clinical and experimental reports should specify DMSO concentration, total dose or volume, route, exposure duration, and formulation.

## 12. Conclusions

Biological effects of DMSO are influenced by concentration and route, whereas the degree of clinical support varies across applications. Intravesical DMSO has the clearest contemporary clinical support in selected patients with IC/BPS, particularly those with Hunner-type disease; however, its benefit in non-Hunner disease and its long-term durability remain uncertain. Most other applications are supported by limited clinical findings or remain experimental. Cardiovascular protection is supported mainly by preclinical studies, and uncontrolled CNS reports of reduced intracranial pressure do not establish lasting neurological benefit. Reports concerning acute CNS trauma, oncology, and ocular or otic applications should be regarded as hypothesis-generating observations rather than established clinical indications. In cryopreservation, DMSO remains an established processing cryoprotectant, whereas reduced-DMSO and DMSO-free alternatives require product-specific assessment and validation under clinical manufacturing conditions. Overall, conclusions about DMSO’s effects and risks remain constrained by exposure conditions, intended application, and methodological limitations. Future studies should specify exposure and formulation, use appropriate comparators, and assess route-specific clinical outcomes and safety.

## Figures and Tables

**Table 1:** Principal quantitative studies of intravesical DMSO for IC/BPS.

Study	n	Design	DMSO Regimen	Comparator	Follow-Up	Primary Outcome	Quantitative Findings	Safety/Main Limitation	Reference
Yoshimura et al., 2021	Randomized/FAS: 96 (49/47); week-12 ICSI: 93 (48/45); week-12 GRA: 94 (48/46)	Multicenter randomized double-blind placebo-controlled trial; DMSO n = 49, placebo n = 47	50 mL of KRP-116D [50% (*w*/*w*) DMSO aqueous solution; 27 g DMSO per 50 mL], every 2 weeks for 12 weeks.	Placebo	12 weeks	Change in ICSI total score	ICSI LS mean change: −5.2 vs. −3.4; difference −1.8 (95% CI, −3.3 to −0.3; *p* = 0.0188). GRA responders: 25/48 vs. 14/46 (*p* = 0.0385).	Predominantly Hunner-type population; 12-week follow-up limits conclusions about sustained benefit.	[29]
Yoshimura et al., 2022; post hoc analysis of the same trial	Hunner-type FAS: 83 (42/41); week-12 GRA: 81 (41/40); non-Hunner: 13	Post hoc phenotype analysis	Same as the parent trial: 50 mL of KRP-116D [50% (*w*/*w*); 27 g DMSO per 50 mL] every 2 weeks for 12 weeks.	Placebo	12 weeks	Post hoc outcomes: symptom changes and GRA	Hunner-type GRA responders: 22/41 (53.7%) vs. 11/40 (27.5%); *p* = 0.0236	Exploratory subgroup analysis; small non-Hunner sample	[30]
Akiyama et al., 2024	30	Single-center retrospective study in refractory Hunner-type IC	50 mL of the approved 50% (*w*/*w*) DMSO formulation containing 27 g DMSO, every 2 weeks for 12 weeks.	None	12 weeks; relapse follow-up	Global response assessment	Response at 12 weeks: 70%; mean relapse-free interval: 6.4 ± 3.9 months	Bladder irritation: 21/30 (70%), resolving within 3 days; no AE-related discontinuations	[31,40]
Moss et al., 2023	83; 70 completed	Prospective randomized trial	50 mL DMSO plus 1 mL triamcinolone (10 mg/mL), weekly for 6 weeks; the DMSO concentration and concentration basis were not specified in the available full text.	Bupivacaine + triamcinolone + heparin	End of treatment	>29.5% reduction in ICSI	Response: 63% vs. 43% (*p* = 0.15); pain and nocturia favored the DMSO regimen	Combination therapy prevents attribution of effects to DMSO alone	[33]
Perez-Marrero et al., 1988	33	Randomized crossover trial	50 mL of 50% DMSO every 2 weeks; two four-treatment periods (concentration basis not specified in the accessible primary report).	Saline	End of each period	Subjective and objective improvement	Marked subjective improvement: 53% vs. 18%; objective improvement: 93% vs. 35%	No significant DMSO-related adverse effects reported	[41,42]
Peeker et al., 2000	21	Randomized double-blind crossover trial; classic (n = 11) and nonulcer (n = 10) IC	50 mL DMSO solution at 500 mg/mL, weekly for 6 weeks.	BCG	3 months after each course	Symptoms and bladder capacity	Pain decreased in both phenotypes (*p* < 0.05); frequency improved only in classic IC; functional capacity did not improve	Small phenotype subgroups limit precision	[42,43]
Tomoe, 2015	28	Retrospective controlled study after hydrodistension; DMSO n = 14, historical control n = 14	50 mL of 50% DMSO in distilled water (concentration basis not specified): weekly ×8, every 2 weeks ×8, then every 4 weeks.	Hydrodistension alone	24 months	Voiding measures and symptom indices	In Hunner-type IC at 24 months: mean voided volume 221 ± 98 vs. 99 ± 32 mL (*p* < 0.02); ICSI 4.2 ± 3.3 vs. 11.6 ± 3.0 (*p* < 0.01)	Benefit was confined to the Hunner-type subgroup; retrospective nonconcurrent comparison	[44]
Cervigni et al., 2017	110 women	Randomized open-label multicenter trial; DMSO n = 36, HA/CS n = 74	RIMSO-50^®^ [50% (*w*/*w*) aqueous DMSO; 50 mL], weekly for 13 weeks.	HA 1.6% + CS 2.0%	6 months	Pain VAS	Per-protocol VAS reduction at 6 months: 28.89 ± 31.14 vs. 44.77 ± 25.07 (*p* = 0.0186)	Any AE: 30.56% vs. 14.86%; treatment-related AE: 22.22% vs. 1.35% (*p* = 0.001)	[45]
Tutolo et al., 2017	36	Prospective randomized trial; DMSO n = 14, CS n = 22	DMSO prepared as a 50% solution in 50 mL physiological saline, weekly for 6 weeks (percentage basis not specified).	CS 2%	End of treatment	Global response assessment	Response: 14% with DMSO vs. 72.7% with CS (*p* = 0.002)	57% of DMSO recipients withdrew; reasons included pain, garlic odor, and lack of efficacy	[46]

Note: Combination regimens do not isolate the effect of DMSO. BPS, bladder pain syndrome; CS, chondroitin sulfate; DMSO, dimethyl sulfoxide; GRA, Global Response Assessment; HA, hyaluronic acid; IC, interstitial cystitis; IC/BPS, interstitial cystitis/bladder pain syndrome; ICSI, Interstitial Cystitis Symptom Index; NR, not reported. Paired values are presented in DMSO/comparator order. Outcome-specific denominators reflect available assessments and may differ from the full analysis set. The post hoc analysis used participants from the parent trial and does not represent an independent sample. FAS, full analysis set; LS, least squares. Concentration bases are stated only when supported by study materials or, for a named marketed formulation, its official label. The 500 mg/mL mass concentration reported for Peeker et al. is retained without conversion to *w*/*w*. Where a report gave only a percentage without defining *w*/*w*, *w*/*v*, or *v*/*v*, the basis is identified as unspecified. Concentrations were not recalculated from component volumes.

**Table 2:** Representative clinical and historical studies of DMSO outside IC/BPS.

Study/Design	Population	DMSO Regimen	Comparator/Follow-Up	Quantitative Findings	Safety/Main Limitation	Reference
Eberhardt et al., 1995; randomized double-blind placebo-controlled phase III trial; active knee gonarthrosis	112 randomized: DMSO n = 56; gel-base placebo n = 56.	25% DMSO gel; 5–8 cm applied three times daily (dose/application details reported in a later systematic review); 3 weeks.	Gel-base placebo; assessments through day 21.	Mean within-group reduction in pain under loading was 42.7 mm (64.5%) with DMSO and 30.8 mm (46.5%) with placebo. The estimated between-group difference in reduction favored DMSO by 11.7 mm (95% CI, 5.1 to 18.35). Primary pain outcomes favored DMSO: loading, *p* = 0.019; rest, *p* = 0.015; palpation, *p* = 0.029.	No serious AEs or laboratory changes reported in the primary abstract.	[50,51,53]
Williams et al., 1985; prospective randomized double-blind multicenter trial; systemic-sclerosis digital ulcers	84 patients across 3 groups; exact group counts NR in the available report.	Topical 2% DMSO or 70% DMSO; application amount and treatment duration NR.	0.85% normal saline; outcomes included ulcer number/area, infection, inflammation and pain.	No significant between-group differences in any stated ulcer or pain outcome; improvement could not be attributed to treatment.	More than one-quarter of patients receiving 70% DMSO withdrew because of significant skin toxicity; exact withdrawal count NR.	[52]
Bookman et al., 2004; randomized double-blind 3-arm trial; primary knee osteoarthritis	248 randomized: diclofenac/DMSO n = 84; DMSO vehicle n = 80; low-DMSO placebo n = 84.	1.5% diclofenac sodium in a 45.5% DMSO carrier; 40 drops (~1.3 mL) four times daily for 4 weeks. Vehicle contained 45.5% DMSO; placebo contained 4.55% DMSO.	DMSO vehicle without diclofenac and placebo solution; 4 weeks.	WOMAC pain mean change −3.9 (95% CI −4.8 to −2.9) vs. −2.5 (−3.3 to −1.7) in both vehicle and placebo (*p* = 0.023 and 0.016).	Skin irritation/dryness occurred in 30/84 (36%) active-formulation patients; treatment discontinuation 5/84 (6%); no serious GI or renal AEs. DMSO effect cannot be separated from diclofenac.	[54]
Roth & Shainhouse, 2004; randomized double-blind vehicle-controlled trial; primary knee osteoarthritis	326 randomized: topical diclofenac/DMSO n = 164; DMSO vehicle n = 162.	1.5% diclofenac sodium in carrier containing 45.5% DMSO; 40 drops (~1.3 mL) applied four times daily for 12 weeks.	Vehicle contained the same carrier (including 45.5% DMSO) without diclofenac; therefore the trial estimates added diclofenac benefit, not DMSO efficacy.	WOMAC pain change −5.9 ± 4.7 vs. −4.3 ± 4.4 (*p* = 0.001); physical function −15.4 ± 15.3 vs. −10.1 ± 13.9 (*p* = 0.002); stiffness −1.8 ± 2.1 vs. −1.3 ± 2.0 (*p* = 0.005); pain on walking −1.18 ± 1.11 vs. −0.87 ± 1.06 (*p* = 0.004).	Minor application-site irritation in 68/164 (41.5%); 5/164 withdrew for skin reactions vs. 1/162 vehicle; reactions resolved after stopping. Safety and efficacy reflect the combination product, not isolated DMSO.	[55]
Leeyaphan et al., 2022; pilot randomized trial; nondermatophyte onychomycosis	20 enrolled (10/arm); 19 evaluable (amphotericin arm n = 10, vehicle n = 9).	Topical 0.3% amphotericin B in 30% DMSO cream vs. 30% DMSO vehicle; once daily before bedtime for 36 weeks.	DMSO-only vehicle; post-treatment follow-up to 72 weeks.	Clinical cure 7/10 (70%) vs. 2/9 (22.2%); mycological cure 8/10 (80%) vs. 4/9 (44.4%).	No adverse events reported. The vehicle arm also showed antifungal activity; small pilot study, not evidence for DMSO as monotherapy across skin indications.	[56]
Marshall et al.; uncontrolled intracranial-hypertension series	6 patients	10% rapid IV bolus (n = 2) or 20% IV titrated to ICP (n = 4)	No comparator; acute ICP monitoring	ICP was initially controlled	All six developed fluid-management or electrolyte problems; standard infusion systems were damaged; no durable neurological endpoint	[57]
Karaca et al.; severe closed-head trauma	10 patients	28% DMSO, 1.12 g/kg IV every 6 h for 1–10 days	Uncontrolled; ICP 40–127 mmHg at baseline	ICP decreased during treatment; report did not establish a controlled long-term neurological effect	Small uncontrolled cohort; systemic exposure and co-interventions limit attribution	[58]

Note: Findings are presented according to study design and exposure context. IV, intravenous; ICP, intracranial pressure; NR, not reported; IC/BPS, interstitial cystitis/bladder pain syndrome.

**Table 4:** Quantitative safety findings across selected DMSO exposure settings.

Exposure Setting	Study/Sample	DMSO Exposure	Quantitative Safety Findings	Clinical Context/Limitation	Source
Intravesical administration	Shimada et al., 2020; six healthy men	50 mL of 50% *w*/*w* DMSO retained for 15 min	Plasma DMSO detected in 1/6; estimated absorption 16.3%; garlic-like breath in 4/6 (66.7%)	Single-dose phase I data indicate limited systemic exposure; sample size was very small	[40]
Historical intravenous administration	Bennett and Muther, 1981; seven patients with stable spinal cord injury; Samoszuk et al., 1983 case report; Marshall et al., 1984 preliminary intracranial-pressure series	IV 10–40% DMSO in Bennett and Muther; IV 10–20% titrated to intracranial pressure in Marshall; exact exposure NR for the Samoszuk case report	Hemoglobinemia and hemoglobinuria occurred in the seven-patient series without a short-term decrease in glomerular filtration rate; severe hemolysis was reported in a separate case; fluid or electrolyte complications occurred in all six Marshall patients; a peak serum osmolality of 717 mOsm/kg was reported separately	Historical, heterogeneous, and uncontrolled exposures indicate route-specific systemic risk but do not define a universal toxicity threshold	[57,113,114,115]
Cryopreserved hematopoietic progenitor-cell infusion	Otrock et al., 2017; 1269 first-day infusions in 1191 adults	DMSO-containing HPC-A products; exact DMSO concentration/dose NR	AEs occurred in 480/1269 infusions (37.8%); among AE infusions, flushing 39.4%, nausea/vomiting 38.1%, hypoxia 29.0%, and chest tightness 16.7%	Larger infusion volume/kg and granulocyte dose/kg predicted AEs; the data do not justify a universal DMSO g/kg threshold	[116]
Topical administration	Roth and Fuller, 2012; pooled safety data from seven blinded phase III trials; 280 patients aged ≥ 75 years	Diclofenac 1.5% in 45.5% DMSO (n = 138), 45.5% DMSO vehicle (n = 103), or 2.33%/4.55% DMSO placebo (n = 39)	Dry skin: 36.2% with diclofenac/DMSO, 18.4% with 45.5% DMSO vehicle, and 2.6% with low-DMSO placebo; DMSO vehicle vs. placebo *p* = 0.0142	Supports a concentration-related local tolerability signal; systemic gastrointestinal, cardiovascular, and renal events were uncommon or not different between groups	[122]

Note: Safety estimates are route-, concentration-, and context-specific. AE, adverse event; DMSO, dimethyl sulfoxide; HPC-A, apheresis-derived hematopoietic progenitor cells.

## Data Availability

No new data were created or analyzed in this review. Data sharing was not applicable to this article.

## Supplementary Materials

The following supporting information can be downloaded at: https://www.mdpi.com/article/10.3390/biom16101534/s1, Table S1: Animal, in vitro, and model studies of DMSO; Table S2: Historical reports and narrative accounts of DMSO therapeutic applications; Table S3: Study appraisal and supporting judgments; Table S4: Search strategies and records identified in PubMed, Web of Science Core Collection, and ClinicalTrials.gov.
