# Supplementary material for: Biological and Clinical Applications of Dimethyl Sulfoxide: Evidence and Safety Considerations
Source: Biomolecules. 2026 Oct 21;16(10):1534. doi: 10.3390/biom16101534 (PMC13702099; doi:10.3390/biom16101534)
Supplement: Supplementary file 1 [file biomolecules-16-01534-s001.zip › biomolecules-4623712 Supplementary Material.pdf]

Table S1. Animal, in vitro, and model studies of DMSO.

Study/model	Sample/model	DMSO exposure	Comparator	Quantitative findings	Safety/main limitation	Reference
Malajczuk et al., 2022; molecular-dynamics simulation of DOPC bilayers	In silico bilayer model; biological n is N/A. Independent simulation count NR.	DMSO–water mixtures at mole fraction xDMSO across dilute (<0.3), intermediate (0.3–0.7), and high (≥0.7) ranges; hydration varied.	DOPC bilayer at full, low, and dehydrated conditions; no biological vehicle arm.	At xDMSO<0.3, modest lateral contraction/surface dehydration; dehydrated-bilayer destabilization at xDMSO≥0.7.	Computational mechanism only; mole fraction is not directly interchangeable with v/v cell-culture concentration. DOI 10.1021/acs.jpcb.2c00353.	[1]
Gallardo-Villagrán et al., 2022; primary human rheumatoid-arthritis fibroblast-like synoviocytes	Cells from 5 women with RA (5 patient-derived samples); assays repeated 3 times. Some initial dose-finding used one donor line.	0.01–10% v/v across assays. Flow cytometry: 0, 2.5, 5, 7.5, and 10% for 24 h in one donor-derived line. Apoptosis-marker assays and MTT: 0.01–5% for 24–72 h.	0% DMSO controls; multiple DMSO concentrations.	At 0.5% for 24 h, MTT indicated approximately 25% cell death. Caspase-3 and PARP-1 cleavage was detected at 5%; flow cytometry showed sharply increased cell death at 5–10%. The authors identified <0.05% as the lowest-impact range at 24 h and 0.01% for longer exposure.	Cell-type and assay-specific toxicity; does not establish a general safe concentration for all cells. PMID 35889344; DOI 10.3390/molecules27144472.	[7]
Amaral et al., 2024; DMPC/DPPC liposome biophysics using DSC/EPR	Model liposomes: three preparation sets for DSC; three independent EPR experiments. Three successive DSC scans/sample were for reproducibility, not independent biological replicates.	DMSO 0–40% v/v for DSC (0, 0.7, 1, 2, 3, 4, 5, 7, 10, 20, 30, 40%); EPR at 0, 10, 20, 30, 40%. Drug–membrane assays used 0–10% DMSO. In vitro model, no organism-level route.	DMSO-free lipid model systems.	With up to 10% DMSO, thermotropic changes were within experimental uncertainty; at 20–40%, transition-temperature shifts and increased fluidity were observed, and at >30% DMSO the DPPC spin probe partly dissociated from bilayers. Three independent EPR experiments.	Model-membrane study; the concentration series and replicate structure limit direct biological interpretation. PMID 38535260; DOI 10.3390/jfb15030067.	[16]
Elisia et al., 2016; ex-vivo human whole blood and mouse serum-transfer arthritis	Human donor number NR; whole-blood assays were triplicates representa-	Human whole blood: 0.25–2% DMSO for cytokine assays and concentrations up to 10% for cell-viability/subtype anal-	No-DMSO human blood; water-dipped mice and non-arthritic controls.	DMSO suppressed cytokines/chemokines/PGE2; monocyte viability began decreasing at 1% and granulocyte/lymphocyte viability at 5–10%. In mice, arthritis swelling was re-	Reduced blood monocytes/lymphocytes are a biological safety signal, not merely an efficacy marker. Model-specific preclinical evidence. PMID 27031833;	[22]

Study/model	Sample/model	DMSO exposure	Comparator	Quantitative findings	Safety/main limitation	Reference
	tive of two independent studies. Arthritis model: 7–9 mice/group.	yses; 15-min preincubation followed by E. coli or HSV-1 stimulation for 7 h. Mice: 70% DMSO/30% water hind-paw immersion, 30 s/paw once, 24 h before arthritis induction; K/BxN serum 100 μL i.p. on days 0 and 2; follow-up to day 7.		duced, with strongest significant effect in forepaws; joint inflammatory markers and circulating WBCs were also reduced.	DOI 10.1371/journal.pone.0152538.	
de la Torre et al., 1975; narrative synthesis of experimental CNS injuries in monkeys, dogs and rats	Multiple animal experiments summarized; pooled n not stated and cannot be treated as one cohort.	Study-specific dose, route and timing vary; not extractable as one exposure from the review.	Various untreated and active-treatment controls across models.	The review reports favorable findings in extradural mass lesions, MCA occlusion, respiratory anoxia and spinal-cord injury, but supplies no single pooled numerical effect estimate.	Secondary synthesis, not one primary experiment; model-level quantitative estimates and toxicity cannot be pooled. PMID 805558; DOI 10.1111/j.1749-6632.1975.tb25377.x.	[59]
de la Torre et al., 1975; experimental permanent spinal-cord injury in dogs	DMSO n=8; dexamethasone n=8; control-arm sample sizes not reported in the accessible abstract and secondary review material.	DMSO dose/concentration not reported in the accessible material; treatment began 1 h after a 500-g·cm cord injury and continued for 3 days.	Dexamethasone, isotonic saline and mannitol; neurological follow-up to 90 days.	Partial or full recovery in 6/8 DMSO-treated dogs vs 2/8 dexamethasone-treated dogs; saline and mannitol were ineffective.	Animal result only; safety outcomes not reported in the accessible material. PMID 1168870; DOI 10.1212/WNL.25.6.508.	[61]
Brown et al., 1980; experimental occipitofrontal missile injury in rhesus monkeys	Rhesus monkeys; group sample sizes not reported in the accessible abstract.	DMSO dose, concentration, route, and timing were not reported in the accessible abstract.	Mannitol-treated and untreated injured animals.	DMSO group had higher blood pressure, cerebral perfusion pressure, cerebral blood flow and oxidative metabolism than both comparator groups; Numerical effect estimates were not reported in the accessible abstract.	Preclinical physiology endpoints; safety and mortality data were not reported in the accessible abstract. PMID 6774065; DOI 10.3171/jns.1980.53.1.0058.	[62]

Study/model	Sample/model	DMSO exposure	Comparator	Quantitative findings	Safety/main limitation	Reference
Phillis et al., 1998; gerbil cerebral ischemia/reperfusion	Gerbil group sizes were not reported in the accessible abstract.	DMSO 2.8 mmol/kg intraperitoneally once, 30 min before ischemia; bilateral carotid occlusion 5 min, reperfusion 5 days.	Saline and ethanol 2.0 mmol/kg.	CA1 pyramidal-neuron loss was reduced with DMSO and ethanol; postischemic locomotor activity increased in all groups, with no between-group differences.	Animal model; Safety endpoints were not reported in the accessible abstract. PMID 9572597; DOI 10.1016/S0304-3940(98)00139-6.	[63]
Bulama et al., 2022; rat traumatic brain injury model	21 rats; 7/group (DMSO-treated TBI, untreated TBI, uninjured control).	67.5 mg/kg orally once daily for 21 days, starting 30 min after injury.	Untreated TBI and uninjured controls.	Improved learning/memory and locomotor outcomes; lower anxiety, S100B and lipid peroxidation; higher SOD, CAT and GPx vs untreated TBI (reported $P<.05$ for biochemical comparisons).	Authors reported no observable toxicity in treated rats. Animal evidence does not establish patient benefit. PMID 36353489; DOI 10.3389/fphar.2022.998179.	[64]
Leon et al., 1970; experimental myocardial necrosis/healing	NR.	Concentration, dose and route NR.	NR.	No numerical outcome was reported.	Quantitative efficacy and safety details were insufficient for extraction. PMID 5413173; DOI:10.1016/0002-8703(70)90425-4.	[65]
Parisi et al., 2010; rat acute myocardial infarction after LAD ligation	Group sizes varied by assay: cardiac damage n=12 control/n=8 DMSO; troponin I n=8/n=4; myoglobin n=7/n=4.	500 µL/kg intraperitoneally daily for 3 days before coronary ligation; DMSO concentration NR.	Untreated infarct controls.	Cardiac damage 18.75±4.88% vs 4.46±2.01% ($P<.05$); troponin I 29.35±12.32 vs 2.95±1.32 ng/mL; myoglobin 46.86±10.35 vs 13.75±0.85 ng/mL (control vs DMSO; assay-specific n above).	Preclinical pretreatment model; Hemodynamic and toxicity outcomes NR. PMID 19904216; DOI 10.1097/FJC.0b013e3181c87a65.	[67]
Dmitriev et al., 2012; isolated rat-heart global ischemia/reperfusion	Rat/isolated-heart sample sizes were not reported in the accessible abstract.	DMSO was given intraperitoneally for 3 days before isolation or added to perfusate/intracoronary perfusion; Exact dose, concentration, and exposure duration were not reported in the accessible abstract.	Untreated ischemic hearts and route-specific DMSO conditions.	Three-day intraperitoneal pretreatment reduced infarct size; intraperitoneal and intracoronary DMSO reduced postischemic LV dysfunction, while DMSO added to perfusate did not reduce infarct size. No numerical effect estimates were reported.	Isolated-organ/animal evidence; safety measures NR. PMID 23330088; DOI 10.1007/s10517-012-1872-8.	[68]

Study/model	Sample/model	DMSO exposure	Comparator	Quantitative findings	Safety/main limitation	Reference
Tunçer & Gurbanov, 2023; E. coli multi-omics study	E. coli model; Biological replicate count NR.	1.0% and 2.5% v/v described as non-growth-inhibitory; 5.0% slightly growth-inhibitory. Exposure duration NR.	Untreated/vehicle-free bacteria and dose comparisons.	At 1.0% and 2.5%, DMSO altered ROS, nucleic-acid content/DNA topology, global 5-methylcytosine pattern, and transcription.	Bacterial biology cannot be directly generalized to mammalian cells; illustrates that "non-growth-inhibitory" is not biologically inert. PMID 36414758; DOI 10.1007/s00253-022-12296-0.	[119]
Gomes et al., 2025; zebrafish embryo developmental toxicity	10 embryos/well in 2 mL; 3 biological replicates, n=30 per treatment concentration.	1–5% v/v from 24 to 72 h post-fertilization; immersion exposure.	E3 medium control; concentration series.	Nearly all embryos exposed to 5% DMSO died after 48 h; 1–4% produced concentration-dependent changes in tail curvature, heart rate/edema, somites, myofibrils, melanocytes, and organ morphology.	Developmental animal model, not a human clinical dose threshold. PMID 40824954; DOI 10.1371/journal.pone.0330348.	[120]

Note: These studies provide mechanistic or preclinical evidence and should not be interpreted as direct evidence of human therapeutic efficacy. CNS, central nervous system; DMSO, dimethyl sulfoxide; NR, not reported; N/A, not applicable.

Table S2. Historical reports and narrative accounts of DMSO therapeutic applications.

Source	Application and material	Source type	Verification status	Reported observation or proposal	Main limitation
Walker's account of William J. Shaal and other acute spinal-cord injury cases [11] (ch. 9, p. 139)	Intravenous DMSO after a reported cervical injury at the University of Oregon Health and Science Center, as described by Walker	Secondary historical case narrative	Walker's account was checked in a digitized copy of the monograph; the underlying clinical record was not independently verified.	Walker described neurological recovery during subsequent care and rehabilitation.	The underlying clinical record and patient-level treatment details were unavailable for verification; concurrent care limits attribution, and the account does not establish efficacy.
Later oncology accounts summarized by Walker [11]	DMSO–haematoxylon treatment in patients with advanced malignancies	Secondary historical narrative	Reported in Walker's monograph; original patient-level reports were not independently verified	Walker described a 37-patient series and reported clinical improvement or tumour regression in some patients	No controlled comparator; heterogeneous malignancies and concomitant treatments; reported responses cannot be independently confirmed
Unpublished 1979 Rogers manuscript cited by Walker [11]	Related DMSO–hematoxylin oncology work, as described by Walker	Unpublished material cited in a secondary source	The manuscript was not independently obtained or examined; information is available only through Walker's citation/account	Walker referred to an unpublished Rogers manuscript concerning related oncology work	Primary document unavailable; study design, methods, population/model, and results cannot be independently assessed
Ophthalmic accounts summarized by Walker [11]	Local DMSO use for corneal swelling and other ocular disorders	Secondary practitioner narratives	Reported in Walker's monograph; underlying practitioner reports or clinical records were not independently verified	Individual observations and treatment proposals were described.	Formulations, comparators, follow-up, and independent verification are incomplete.
Chronic tinnitus account summarized by Walker [11]	DMSO-containing otic/external-ear preparations for chronic tinnitus	Secondary historical narrative	Reported in Walker's monograph; original report, patient records, and complete formulation details were not independently verified	Walker reported complete symptom disappearance in 9/15 patients, substantial improvement in 2, and altered but persistent symptoms in 4	Small uncontrolled series; formulation incompletely characterized; outcomes and source data were not independently verified
Tucker and Carrizo [71]	DMSO–hematoxylin preparation, termed "haematoxylon" in the	Published historical article	The title page and materials and methods section of a scanned original article were examined. The description of the crystalline	The report described hematoxylin dissolved in DMSO for recurrent neoplasms.	Patient-level outcomes and the complete treatment protocol were not verified in this check.

Source	Application and material	Source type	Verification status	Reported observation or proposal	Main limitation
	original report, for recurrent neoplasms		material and its molecular formula support identification as hematoxylin.		
Rogers [72]	DMSO–hematoxylin in a transplantable lymphosarcoma	Archival doctoral dissertation; experimental work	The 1970 North Texas State University doctoral dissertation was independently identified through the university catalogue; its relationship to the unpublished 1979 manuscript cited by Walker could not be established.	Investigated the combined formulation in a tumour model.	Not a human clinical observation. The 1970 doctoral dissertation has not been established as the same document as the unpublished 1979 manuscript cited by Walker; the two should not be treated as one study.
Hill [76]	DMSO treatment in retinal disease	Published original clinical report	The PubMed and publisher bibliographic records were verified. The numerical report was available through a secondary web reproduction of the article text; the publisher full text was not accessible for this check.	In 50 patients with retinal degeneration, improvements were reported in visual acuity, visual fields, and dark adaptation in 22, 9, and 5 patients, respectively	Historical uncontrolled report; DMSO concentration was not provided in the accessible secondary reproduction; no controlled comparator

Note: The observations listed here describe historical sources and should not be regarded as independently verified treatment effects. The Rogers dissertation concerns an experimental tumour model, not a human case. CNS, central nervous system; DMSO, dimethyl sulfoxide.

Table S3. Study appraisal and supporting judgments.

Study / reference	Design and result appraised	Tool, version, and source access	Domain-level judgment	Overall judgment or design-specific limitation	Judgment rationale and effect on interpretation	Supporting location
Randomized clinical studies						
Yoshimura et al., 2021 [29]	Parallel-group RCT; week-12 change in ICSI total score.	RoB 2, parallel-trial version 22 Aug 2019; full text (PMCID: PMC8247858).	D1 Low; D2 Low; D3 Low; D4 Low; D5 Low.	Low risk for this result.	Central dynamic allocation and independent sequence generation were reported. Patients, investigators, and the sponsor were masked, and identical vials were used. The full-analysis set and a prespecified mixed-effects model were applied. These features support a low risk of bias for the stated result, although sponsor involvement in trial design and analysis should be considered when interpreting the modest between-group difference.	Full text: Methods - Study design and participants; Randomization and treatment; Statistical analysis; Results - Table 2 and Supplementary Table S2.

Study / reference	Design and result appraised	Tool, version, and source access	Domain-level judgment	Overall judgment or design-specific limitation	Judgment rationale and effect on interpretation	Supporting location
Moss et al., 2023 [33]	Open-label parallel-group RCT; primary outcome: proportion of patients with a >29.5% reduction in total ICSI score after six weekly instillations.	RoB 2, parallel-trial version 22 Aug 2019; full text (DOI: 10.1002/nau.25142; PMID: 36747494).	D1 Some concerns; D2 Some concerns; D3 High; D4 High; D5 Some concerns.	High risk of bias for the primary patient-reported ICSI outcome.	The trial was open-label, and the primary outcome was patient-reported. Of 83 randomized participants, 70 completed the six weekly instillations and entered the final analysis (42 in the DMSO group and 28 in the BTH group). Missing outcome data and lack of masking may have affected the treatment comparison; the between-group difference was not statistically significant (P = 0.15).	Full text: Materials and Methods—Study design and outcome measures; Results—participant flow and Tables 2–4.

Study / reference	Design and result appraised	Tool, version, and source access	Domain-level judgment	Overall judgment or design-specific limitation	Judgment rationale and effect on interpretation	Supporting location
Perez-Marrero et al., 1988 [41]	Randomized crossover trial; subjective and objective improvement after each treatment period.	RoB 2 crossover version 18 Mar 2021; abstract only (PMID: 3288775).	D1 Some concerns; D2 Some concerns; D3 Some concerns; D4 Some concerns; D5 Some concerns; S: Some concerns.	Provisional some concerns; full appraisal not possible from the abstract.	Random allocation and saline control were reported, but sequence generation, allocation concealment, washout adequacy, and period/carryover effects were insufficiently described. Missing-data handling and a prespecified analysis plan were also unavailable. DMSO-related sensory cues may additionally have affected masking.	PubMed abstract; page-level location unavailable.

Study / reference	Design and result appraised	Tool, version, and source access	Domain-level judgment	Overall judgment or design-specific limitation	Judgment rationale and effect on interpretation	Supporting location
Peeker et al., 2000 [43]	Randomized double-blind crossover trial; symptoms and bladder capacity at three months.	RoB 2 crossover version 18 Mar 2021; abstract and bibliographic record (PMID: 11061879).	D1 Some concerns; D2 Some concerns; D3 Some concerns; D4 Some concerns; D5 Some concerns; S: Some concerns.	Provisional some concerns; full domain-level appraisal was limited by incomplete access to the primary report.	The report identifies randomization and double blinding, but the accessible material does not adequately describe allocation concealment, attrition handling, washout adequacy, or period/carryover effects. The small sample further limits precision.	PubMed abstract; page-level location unavailable.

Study / reference	Design and result appraised	Tool, version, and source access	Domain-level judgment	Overall judgment or design-specific limitation	Judgment rationale and effect on interpretation	Supporting location
Cervigni et al., 2017 [45]	Open-label parallel RCT; change in pain VAS at six months.	RoB 2, parallel-trial version 22 Aug 2019; PubMed abstract and publisher record (PMID: 27654012; DOI: 10.1002/nau.23091).	D1 Some concerns; D2 Some concerns; D3 Some concerns; D4 High; D5 Some concerns.	Provisional high risk for the patient-reported pain result; full domain-level confirmation was limited by the absence of the complete report.	The report describes the study as randomized, but the accessible record does not specify sequence generation or allocation concealment. The study was open label, and the primary pain outcome was patient reported. Per-protocol analysis and differential tolerability may also have affected the treatment comparison.	PubMed abstract and publisher record; the accessible record does not provide page-level details on sequence generation or allocation concealment.

Study / reference	Design and result appraised	Tool, version, and source access	Domain-level judgment	Overall judgment or design-specific limitation	Judgment rationale and effect on interpretation	Supporting location
Tutolo et al., 2017 [46]	Parallel RCT; GRA response after six weekly instillations.	RoB 2, parallel-trial version 22 Aug 2019; full text (PMCID: PMC5293394).	D1 Some concerns; D2 High; D3 High; D4 High; D5 Some concerns.	High risk.	No masking was reported, the outcome was patient reported, and withdrawal was 8/14 (57%) with DMSO versus 6/22 (27%) with chondroitin sulfate. The large differential dropout directly threatens the reported response comparison.	Full text: Materials and Methods; Results; Table 3 (withdrawals and response).
Eberhardt et al., 1995 [50]	Double-blind parallel RCT; pain under loading at day 21.	RoB 2, parallel-trial version 22 Aug 2019; English abstract plus later systematic-review extraction (PMID: 8529985).	D1 Some concerns; D2 Some concerns; D3 Some concerns; D4 Some concerns; D5 Some concerns.	Provisional some concerns; German full text was not available for this appraisal.	Randomization and double blinding were reported, but allocation concealment, complete attrition data, and a protocol/statistical analysis plan could not be verified from the accessible material.	English-language PubMed abstract and systematic-review extraction; German full-text page location not verified.

Study / reference	Design and result appraised	Tool, version, and source access	Domain-level judgment	Overall judgment or design-specific limitation	Judgment rationale and effect on interpretation	Supporting location
Williams et al., 1985 [52]	Three-arm double-blind RCT; digital-ulcer and pain outcomes.	RoB 2, parallel-trial version 22 Aug 2019; abstract only (PMID: 3884019).	D1 Some concerns; D2 Some concerns; D3 Some concerns; D4 Some concerns; D5 Some concerns.	Provisional some concerns; full domain-level appraisal was limited by the absence of the complete report.	The abstract reports randomization and blinding but not the allocation method, group sizes, missing-data handling, or outcome hierarchy. More than one-quarter of the 70% DMSO group withdrew because of skin toxicity.	PubMed abstract; page-level location unavailable.
Bookman et al., 2004 [54]	Three-arm double-blind RCT; WOMAC pain at four weeks and local safety.	RoB 2, parallel-trial version 22 Aug 2019; full text (PMCID: PMC509045).	D1 Some concerns; D2 Low; D3 Low; D4 Low; D5 Some concerns.	Some concerns.	Intent-to-treat analysis and blinded matched solutions reduce bias. However, the trial was designed to test diclofenac; comparing the 45.5% DMSO vehicle with the low-DMSO placebo does not isolate a prespecified therapeutic effect of DMSO.	Full text: Patients and Methods - Study design/treatments and efficacy measures; Results; Tables 2-4.

Study / reference	Design and result appraised	Tool, version, and source access	Domain-level judgment	Overall judgment or design-specific limitation	Judgment rationale and effect on interpretation	Supporting location
Roth and Shainhouse, 2004 [55]	Double-blind vehicle-controlled RCT; WOMAC outcomes and application-site reactions at 12 weeks.	RoB 2, parallel-trial version 22 Aug 2019; full text (PMID: 15477437).	D1 Low; D2 Low; D3 High; D4 Low; D5 Some concerns.	High risk of bias, driven by missing outcome data and baseline-value imputation for the stated WOMAC result.	Both groups received the same DMSO-containing carrier, so the randomized contrast estimates added diclofenac benefit rather than DMSO efficacy. Missing outcome data and the use of baseline-value imputation remain important concerns for the reported WOMAC result.	Full text: Methods—randomization and allocation concealment; treatment packaging and blinding; WOMAC outcome assessment; Results—withdrawals and missing-data handling.

Study / reference	Design and result appraised	Tool, version, and source access	Domain-level judgment	Overall judgment or design-specific limitation	Judgment rationale and effect on interpretation	Supporting location
Leeyaphan et al., 2022 [56]	Double-blind pilot RCT; mycological cure at 36 weeks.	RoB 2, parallel-trial version 22 Aug 2019; full-text PDF; ClinicalTrials.gov NCT03814343.	D1 Some concerns; D2 Low; D3 Low; D4 Low; D5 Low.	Some concerns.	Mixed-block randomization, identical tubes, blinded clinical assessors, registration, and objective culture/KOH outcomes were reported. Allocation concealment was not described and the study was very small (20 randomized; 19 analyzed), limiting precision rather than automatically creating bias.	Article pp. 495-496: Materials and Methods - Study design; Table 1; trial registry NCT03814343.

Study / reference	Design and result appraised	Tool, version, and source access	Domain-level judgment	Overall judgment or design-specific limitation	Judgment rationale and effect on interpretation	Supporting location
Mitrus et al., 2018 [88]	Three-arm randomized study; time to engraftment and infusion adverse events.	RoB 2, parallel-trial version 22 Aug 2019; abstract only (PMID: 29269805).	D1 Some concerns; D2 Some concerns; D3 Some concerns; D4 Some concerns; D5 Some concerns.	Provisional some concerns; full domain-level appraisal was limited by the available abstract.	The abstract reports random assignment of 150 patients; 143 underwent transplantation and four died before engraftment. Sequence generation, concealment, masking, and prespecified analysis details were not available. Engraftment timing is relatively objective, but the accessible abstract did not provide sufficient detail on the recovery threshold, measurement frequency, or outcome-assessment procedures.	PubMed abstract, Methods and Results; page-level location unavailable.
Non-randomized or uncontrolled clinical studies						

Study / reference	Design and result appraised	Tool, version, and source access	Domain-level judgment	Overall judgment or design-specific limitation	Judgment rationale and effect on interpretation	Supporting location
Akiyama et al., 2024 [31]	Retrospective uncontrolled series; GRA response at 12 weeks and relapse-free interval.	Design-specific appraisal; PubMed/publisher record (DOI: 10.1111/iju.15320).	Selection: convenience/refractory Hunner-type cohort; comparator: none; confounding/co-interventions: not controlled; outcome: partly patient reported; follow-up: limited.	Very limited basis for causal inference; ROBINS-I was not applied because there was no comparison group.	The observed 70% response cannot distinguish treatment effect from regression to the mean, natural fluctuation, placebo response, or concurrent care.	PubMed/publisher abstract, Methods and Results; page-level location unavailable in the accessible record.
Stav et al., 2012 [32]	Uncontrolled cohort using a multi-agent intravesical cocktail; symptom response.	Design-specific appraisal; PubMed record (PMID: 22748865).	Selection: unclear; comparator: none; co-interventions: DMSO, hydrocortisone, heparin sulfate, and bupivacaine; outcome: patient reported; missing data/reporting: incompletely described.	Very limited basis for DMSO-specific causal inference.	Any improvement may arise from the other active components, catheter-related effects, natural symptom variation, or expectancy. The study cannot isolate DMSO.	PubMed abstract, Methods and Results; page-level location unavailable.

Study / reference	Design and result appraised	Tool, version, and source access	Domain-level judgment	Overall judgment or design-specific limitation	Judgment rationale and effect on interpretation	Supporting location
Tomoe, 2015 [44]	Retrospective DMSO cohort with nonconcurrent historical control; 24-month ICSI and voided volume.	ROBINS-I 2016; accessible full-text article/record (PMID: 26816859).	Confounding Serious; selection Serious; classification Low; deviations Moderate; missing data No information; measurement Serious; selective reporting No information.	Serious risk of bias.	The nonconcurrent historical comparison is highly susceptible to confounding by phenotype, disease severity, calendar time, and co-interventions. The patient-reported outcomes were not assessed under blinded conditions. These concerns are sufficient to support an overall Serious judgment. Information on deviations from intended intervention, missing-data handling, and selective reporting was insufficient to justify uniformly Serious judgments in those domains.	Full text: Materials and Methods; Results; tables reporting ICSI and voided-volume outcomes.

Study / reference	Design and result appraised	Tool, version, and source access	Domain-level judgment	Overall judgment or design-specific limitation	Judgment rationale and effect on interpretation	Supporting location
Marshall et al., 1984 [57]	Uncontrolled six-patient series; acute intracranial-pressure response.	Design-specific appraisal; abstract/record (PMID: 6462399).	Selection unclear; no comparator; variable dosing; intensive-care co-interventions; acute surrogate outcome; complete long-term neurological follow-up not established.	Very limited basis for causal inference.	An acute fall in intracranial pressure does not establish durable neurological benefit. All six patients developed fluid-management or electrolyte problems, so safety signals should not be minimized.	PubMed abstract; page-level location unavailable.
Karaca et al., 1991 [58]	Uncontrolled ten-patient severe-head-trauma report; intracranial pressure during treatment.	Design-specific appraisal; abstract/record (PMID: 2060538).	Selection unclear; no comparator; repeated systemic dosing; co-interventions not separable; outcome is an acute surrogate; long-term outcomes inadequately reported.	Very limited basis for causal inference.	The before-after decline in intracranial pressure may reflect concurrent trauma care, natural course, measurement timing, or DMSO. It cannot support a controlled long-term treatment effect.	PubMed abstract; page-level location unavailable.

Study / reference	Design and result appraised	Tool, version, and source access	Domain-level judgment	Overall judgment or design-specific limitation	Judgment rationale and effect on interpretation	Supporting location
Olver et al., 1988 [83]	Prospective uncontrolled single-arm pilot study; ulceration, surgical intervention, residual local injury, and local adverse effects through three months.	Design-specific appraisal; publisher abstract and PubMed record (PMID: 3183703; DOI: 10.1200/JCO.1988.6.11.1732).	Selection: consecutive recruitment not established; comparator: none; co-interventions: not described in the accessible abstract; outcome ascertainment: serial examinations and photographs; missing outcome data: 16/20 completed three-month observation; reporting: no protocol available.	Very limited basis for DMSO-specific causal inference.	No patient progressed to ulceration or surgery, but the uncontrolled design cannot separate DMSO from injury severity, natural course, timing of treatment, or other care. Two patients died before three months and two were lost to follow-up; later tissue injury was therefore not fully observed.	J Clin Oncol 1988;6:1732-1735: publisher abstract (Methods and Results); full-text page-level passages not accessible.

Study / reference	Design and result appraised	Tool, version, and source access	Domain-level judgment	Overall judgment or design-specific limitation	Judgment rationale and effect on interpretation	Supporting location
Bertelli et al., 1995 [84]	Open prospective uncontrolled study; ulceration and local adverse effects after cytotoxic-drug extravasation.	Design-specific appraisal; PubMed abstract and publisher record (PMID: 7595748; DOI: 10.1200/JCO.1995.13.11.2851).	Selection: all institutional extravasations were considered, but inclusion/assessment losses remain incompletely explained; comparator: none; co-intervention: intermittent local cooling for one hour three times daily on days 1-3; clinical heterogeneity: eight cytotoxic agents; missing outcome data: 127/144 assessable; reporting: no protocol available.	Very limited basis for DMSO-specific causal inference.	Only one ulceration was reported, but local cooling prevents attribution of the result to DMSO alone. Variation in extravasated drug and injury characteristics, together with 17 non-assessable patients, further limits causal interpretation.	J Clin Oncol 1995;13:2851-2855: PubMed abstract - Patients and methods; Results; full-text page-level passages not accessible.
Gokarn et al., 2023 [89]	Uncontrolled cryopreserved-graft series; post-thaw CD34+ recovery and viability.	Design-specific appraisal; PubMed record (PMID: 37678607).	Selection unclear; no concurrent comparator; heterogeneous grafts/storage; laboratory endpoints; small sample; clinical outcomes limited.	Useful process feasibility evidence, not comparative efficacy evidence.	Recovery and viability after long storage show feasibility under the reported protocol, but cannot establish equivalence to another cryoprotectant or DMSO concentration.	PubMed abstract, Methods and Results; page-level location unavailable.

Study / reference	Design and result appraised	Tool, version, and source access	Domain-level judgment	Overall judgment or design-specific limitation	Judgment rationale and effect on interpretation	Supporting location
Panch et al., 2019 [90]	Observational manufacturing analysis across CAR-T products; viability, phenotype, persistence, and response.	Design-specific appraisal; full-text/record (PMID: 31178392).	Product selection and trial heterogeneity; no randomized cryopreservation comparator; multiple manufacturing steps; clinical response confounded by product and patient factors.	Moderate value for process description; limited basis for causal inference regarding clinical effects of DMSO.	Similar final expansion and response do not prove that cryopreservation has no effect, because product characteristics and clinical populations were not randomized by preservation condition.	Full text: Methods - Cryopreservation/thawing and product characterization; Results - viability, expansion, persistence, and response figures/tables.
Animal intervention studies						
Elisia et al., 2016 [22]	Mouse serum-transfer arthritis experiment; paw swelling and inflammatory markers.	SYRCLE 2014; full text (PMCID available through PLOS; PMID: 27031833).	Sequence Unclear; baseline Unclear; concealment Unclear; random housing Unclear; caregiver blinding Unclear; random outcome assessment Unclear; assessor blinding Unclear; incomplete data Unclear; selective reporting Unclear; other bias Unclear.	No numerical summary score; reporting leaves most domains unclear.	Group sizes (7-9) and controls were reported, but the accessible report did not establish allocation concealment, housing randomization, or blinded outcome assessment. The human whole-blood component is ex vivo and is appraised separately from the mouse experiment.	Full text: Materials and Methods - K/BxN serum-transfer arthritis and treatment groups; Results - paw swelling and inflammatory markers.

Study / reference	Design and result appraised	Tool, version, and source access	Domain-level judgment	Overall judgment or design-specific limitation	Judgment rationale and effect on interpretation	Supporting location
de la Torre et al., 1975 [61]	Dog permanent spinal-cord injury experiment; neurological recovery to 90 days.	SYRCLE 2014; PubMed record and secondary/full-text review material (PMID: 1168870).	Sequence Unclear; baseline Unclear; concealment Unclear; random housing Unclear; caregiver blinding Unclear; random outcome assessment Unclear; assessor blinding Unclear; incomplete data Unclear; selective reporting Unclear; other bias Unclear.	No overall score; risk remains largely unclear because key methods are not reported.	DMSO and dexamethasone groups each had eight dogs, but control-arm sizes, dose/concentration, masking, and attrition were not adequately documented in the accessible material.	PubMed abstract and secondary/full-text review material; primary full-text page location not verified.
Brown et al., 1980 [62]	Rhesus-monkey missile-injury experiment; physiological endpoints.	SYRCLE 2014; abstract/record (PMID: 6774065).	All ten SYRCLE domains Unclear from the accessible record.	No overall score; full-text appraisal not possible.	Group sizes, DMSO dose/route/timing, numerical effect estimates, mortality, and safety outcomes were not available. These omissions preclude a defensible low-risk judgment.	PubMed abstract; page-level location unavailable.

Study / reference	Design and result appraised	Tool, version, and source access	Domain-level judgment	Overall judgment or design-specific limitation	Judgment rationale and effect on interpretation	Supporting location
Phillis et al., 1998 [63]	Gerbil ischemia/reperfusion experiment; CA1 neuronal loss at five days.	SYRCLE 2014; abstract/record (PMID: 9572597).	Sequence Unclear; baseline Unclear; concealment Unclear; random housing Unclear; caregiver blinding Unclear; random outcome assessment Unclear; assessor blinding Unclear; incomplete data Unclear; selective reporting Unclear; other bias Unclear.	No overall score; largely unclear.	The dose, timing, and comparators were identifiable, but group sizes and bias-control methods were not. The study supports only model-specific preclinical inference.	PubMed abstract; page-level location unavailable.
Bulama et al., 2022 [64]	Rat traumatic-brain-injury experiment; behavioral and biochemical outcomes.	SYRCLE 2014; full text (PMCID: PMC9638698).	Sequence Unclear (stated random, method absent); baseline Low; concealment Unclear; random housing Unclear; caregiver blinding Unclear; random outcome assessment Unclear; assessor blinding Unclear; incomplete data High; selective reporting Unclear; other bias Unclear.	No overall score; attrition/reporting concern.	Seven rats/group were assigned, but several biochemical figures report n=5/group without a clear attrition explanation. Blinding and concealment were not reported. Small groups and many outcomes increase imprecision and selective-reporting risk.	Full text: Materials and Methods - Experimental design; Results - behavioral data and Figures 6-10.

Study / reference	Design and result appraised	Tool, version, and source access	Domain-level judgment	Overall judgment or design-specific limitation	Judgment rationale and effect on interpretation	Supporting location
Parisi et al., 2010 [67]	Rat myocardial-infarction pretreatment experiment; infarct damage and biomarkers.	SYRCLE 2014; abstract/record (PMID: 19904216).	Sequence Unclear; baseline Unclear; concealment Unclear; random housing Unclear; caregiver blinding Unclear; random outcome assessment Unclear; assessor blinding Unclear; incomplete data Unclear; selective reporting Unclear; other bias Unclear.	No overall score; incomplete-data risk is unclear from the accessible record.	Assay-specific sample sizes differed (for example, 12/8 for damage and 8/4 for troponin), but the accessible abstract did not establish whether the assays used prespecified subsets, exclusions, or missing observations. The difference in sample numbers alone was therefore not treated as evidence of high attrition bias. Pretreatment before induced infarction remains a separate limitation on clinical translation.	PubMed abstract, Methods and Results; page-level location unavailable.

Study / reference	Design and result appraised	Tool, version, and source access	Domain-level judgment	Overall judgment or design-specific limitation	Judgment rationale and effect on interpretation	Supporting location
Dmitriev et al., 2012 [68]	Rat/isolated-heart ischemia-reperfusion experiments; infarct size and ventricular function.	SYRCLE 2014; abstract/record (PMID: 23330088).	All ten SYRCLE domains Unclear from the accessible record.	No overall score; full-text appraisal not possible.	Sample sizes, exact dose/concentration, randomization, blinding, and numerical effect estimates were not available. The result should remain a qualitative preclinical signal.	PubMed abstract; page-level location unavailable.
Gomes et al., 2025 [120]	Zebrafish developmental-toxicity experiment; mortality and morphology from 24-72 hpf.	SYRCLE 2014; applied to the zebrafish embryo intervention study where applicable; full text (PMCID/PLOS article; PMID: 40824954).	Sequence Unclear; baseline Low; concealment Unclear; random housing Unclear; caregiver blinding Unclear; random outcome assessment Unclear; assessor blinding Unclear; incomplete data Low for mortality; selective reporting Unclear; other bias Unclear.	No overall score; several bias-control methods are unreported.	Three biological replicates and 30 embryos/concentration were reported, but assignment, blinding, and preregistered outcome selection were not established. The model defines developmental hazard under immersion exposure, not a human therapeutic threshold.	Full text: Materials and Methods - Embryo exposure and developmental assessment; Results - mortality and morphological endpoints.
In vitro, ex vivo, and computational studies						

Study / reference	Design and result appraised	Tool, version, and source access	Domain-level judgment	Overall judgment or design-specific limitation	Judgment rationale and effect on interpretation	Supporting location
Malajczuk et al., 2022 [1]	Molecular-dynamics simulation of DOPC bilayers.	Design-specific reproducibility appraisal; original article (DOI: 10.1021/acs.jpcb.2c00353).	Model specification partly reported; independent simulation count not reported; sensitivity to force field and hydration conditions; no biological randomization or blinding domains apply.	Mechanistic plausibility only.	Mole fraction in a simulation is not interchangeable with a clinical or cell-culture v/v concentration. Conclusions depend on model assumptions and require experimental corroboration.	Original article: Methods/Computational details; Results - membrane structural analyses; exact page location not independently verified.
Gallardo-Villagrán et al., 2022 [7]	Primary human RA fibroblast-like synoviocytes; viability/apoptosis across DMSO concentrations.	Design-specific in-vitro appraisal; full text (PMID: 35889344; DOI: 10.3390/molecules27144472).	Five donor-derived samples; some dose-finding used one donor line; vehicle controls present; assay replication reported; allocation/blinding and prespecified analysis not reported.	Moderate mechanistic value; limited generalizability.	Donor and assay dependence prevents a universal safe-concentration claim. MTT and apoptosis markers strengthen internal consistency, but one-donor dose-finding and small biological n limit inference.	Full text: Materials and Methods - primary RA-FLS culture, MTT, and apoptosis assays; Results - concentration-response figures.

Study / reference	Design and result appraised	Tool, version, and source access	Domain-level judgment	Overall judgment or design-specific limitation	Judgment rationale and effect on interpretation	Supporting location
Amaral et al., 2024 [16]	DMPC/DPPC liposome DSC/EPR experiments.	Design-specific in-vitro/model appraisal; full text (PMID: 38535260; DOI: 10.3390/jfb15030067).	DMSO-free controls and concentration series present; three independent EPR experiments; successive DSC scans are technical reproducibility, not independent biological replicates; blinding not applicable/reported.	Mechanistic model evidence only.	The study supports concentration-dependent membrane effects in simplified lipid systems but cannot establish cellular toxicity or clinical benefit.	Full text: Materials and Methods - DSC/EPR; Results - concentration-dependent membrane effects and associated figures.
Liu et al., 2010 [91]	Controlled in-vitro cryopreservation study of human bone-marrow MSCs; post-thaw viability and differentiation.	Design-specific in-vitro appraisal; abstract/record (PMID: 20572296).	Concurrent 10% DMSO reference present; formulation comparisons reported; donor number, allocation, blinding, and complete replicate structure not confirmed from accessible material.	Useful formulation comparison; final bias judgment requires full methods.	Similar viability under selected formulations does not establish clinical equivalence; donor variability, batch effects, and long-term functional outcomes remain relevant.	PubMed abstract, Methods and Results; page-level location unavailable.

Study / reference	Design and result appraised	Tool, version, and source access	Domain-level judgment	Overall judgment or design-specific limitation	Judgment rationale and effect on interpretation	Supporting location
Tunçer and Gurbanov, 2023 [119]	E. coli multi-omics dose-response study.	Design-specific in-vitro appraisal; abstract/record (PMID: 36414758).	Vehicle-free controls and dose comparisons reported; biological replicate count and exposure duration not available in the extracted record; multiple omics endpoints raise multiplicity concerns.	Mechanistic signal with limited mammalian relevance.	The study shows that non-growth-inhibitory DMSO concentrations can alter bacterial biology. It should not be generalized directly to mammalian cells or therapeutic exposure.	PubMed abstract; page-level location unavailable.
Historical reports and narrative sources						
Walker monograph and cited patient narratives [11]	Secondary historical narratives of CNS, oncology, ocular, and otic use.	Source-authenticity and design-specific appraisal; monograph available, underlying records generally unavailable.	Patient selection unknown; no controlled comparator; co-interventions incompletely documented; outcomes not independently measured; selective reporting cannot be excluded; source accessibility limited.	Anecdotal/hypothesis-generating evidence only.	These accounts can document historical claims but cannot establish efficacy. Neurological recovery must be separated from the two small clinical ICP series and from concurrent intensive care and rehabilitation.	Walker's account of William J. Shaal: Chapter 9, p. 139 of the digitized monograph was examined; the underlying clinical record was unavailable. Other Walker narratives were not independently verified at page level.

Study / reference	Design and result appraised	Tool, version, and source access	Domain-level judgment	Overall judgment or design-specific limitation	Judgment rationale and effect on interpretation	Supporting location
Walker chronic-tinnitus account [11]	Secondary account of a 15-patient uncontrolled series.	Source-authenticity appraisal; original report and patient records unavailable.	Selection unknown; formulation incompletely characterized; no comparator; subjective outcomes; follow-up and adverse-event reporting unavailable.	Unverified anecdotal evidence.	The numerical response account should be presented only as Walker's report, not as independently verified treatment evidence.	Walker monograph tinnitus narrative; original report/patient records and stable page-level location unavailable.
Tucker and Carrizo, 1968 [71]	Published historical report of DMSO-hematoxylin for recurrent neoplasms.	Design-specific appraisal; scanned title page and methods material reported as examined (PMID: 4173033).	No controlled comparator confirmed; heterogeneous malignancies/treatments; patient-level outcomes and full protocol not verified; outcome ascertainment and follow-up unclear.	Major design and reporting limitations.	The source supports that the formulation was investigated, not that antitumor efficacy was established. The spelling distinction between hematoxylin and the historical term haematoxylon should be retained.	Scanned article: title/methods material; patient-level results pages were not verified.

Study / reference	Design and result appraised	Tool, version, and source access	Domain-level judgment	Overall judgment or design-specific limitation	Judgment rationale and effect on interpretation	Supporting location
Rogers dissertation, 1970 [72]	Archival experimental work on DMSO-hematoxylin in transplantable lymphosarcoma.	Design-specific experimental appraisal; university repository record (https://digital.library.unt.edu/ark:/67531/metadc164389/).	Experimental model rather than human clinical study; relationship to Walker's cited unpublished 1979 manuscript not established; bias-control methods require direct dissertation review.	Preclinical historical source; not a human efficacy report.	The 1970 dissertation and the unpublished 1979 manuscript must not be merged as one study without documentary evidence.	University of North Texas repository record; exact methods/results page locations were not independently verified.
Hill, 1975 [76]	Uncontrolled clinical report in 50 patients with retinal degeneration.	Design-specific appraisal; bibliographic record and available report summary (DOI: 10.1111/j.1749-6632.1975.tb25391.x).	Selection unclear; no comparator; DMSO concentration incompletely reported; several outcomes without a prespecified hierarchy; masking and follow-up unclear.	Major design and reporting limitations.	Reported improvements in visual acuity, fields, or dark adaptation cannot be separated from measurement variability, natural history, or selective outcome reporting.	Bibliographic record and available report summary; primary full-text page location not verified.

Note: The appraisal included original intervention studies and historical primary or secondary sources contributing to the principal efficacy, safety, or mechanistic conclusions of the review. Guidelines and reviews were not appraised as intervention studies. RoB 2 [12] judgments apply to the stated result and time point; ROBINS-I 2016 [13] was used only for comparative non-randomized intervention studies, and SYRCLE [14] was used only for animal intervention studies. Uncontrolled, in vitro, computational, and historical sources were assessed descriptively according to design-specific limitations. No cross-tool numerical score was calculated. Judgments based only on abstracts or bibliographic records were treated as provisional because full domain-level appraisal was not possible without the complete report. Risk-of-bias assessments were performed by one author and checked by a second author; they were not independently duplicated. Abbreviations: D1-D5, RoB 2 domains; S, bias arising from period and carryover effects in crossover trials; GRA, Global Response Assessment; ICSI, Interstitial Cystitis Symptom Index; hpf, hours post-fertilization; Low, low risk of bias; High, high risk of bias; Unclear, insufficient reporting.

Supplementary Table S4. Search strategies and records identified in PubMed, Web of Science Core Collection, and ClinicalTrials.gov

Database	Search Step	Direct-search format	Records
PubMed	S1. Core A	(("Dimethyl Sulfoxide"[Mesh] OR "dimethyl sulfoxide"[tiab] OR "dimethyl sulphoxide"[tiab] OR dimethylsulfoxide[tiab] OR dimethylsulphoxide[tiab] OR DMSO[tiab] OR Me2SO[tiab])) AND 1960/01/01:2026/10/12[dp]	44,730
PubMed	S2. A AND B1	(("Dimethyl Sulfoxide"[Mesh] OR "dimethyl sulfoxide"[tiab] OR "dimethyl sulphoxide"[tiab] OR dimethylsulfoxide[tiab] OR dimethylsulphoxide[tiab] OR DMSO[tiab] OR Me2SO[tiab]) AND ("Cystitis, Interstitial"[Mesh] OR "interstitial cystitis"[tiab] OR "bladder pain syndrome"[tiab] OR "painful bladder syndrome"[tiab] OR "IC/BPS"[tiab] OR "IC/PBS"[tiab] OR "Hunner lesion"[tiab] OR "Hunner lesions"[tiab] OR "Hunner disease"[tiab:~0] OR intravesical[tiab] OR "bladder instillation"[tiab] OR "bladder instillations"[tiab] OR "KRP-116D"[tiab])) AND 1960/01/01:2026/10/12[dp]	222

PubMed	S3. A AND B3-biomedical AND cryopreservation-focus	(("Dimethyl Sulfoxide"[Mesh] OR "dimethyl sulfoxide"[tiab] OR "dimethyl sulphoxide"[tiab] OR dimethylsulfoxide[tiab] OR dimethylsulphoxide[tiab] OR DMSO[tiab] OR Me2SO[tiab]) AND (("Cryopreservation"[Mesh] OR cryopreservation[tiab] OR cryopreserved[tiab] OR cryoprotectant[tiab] OR cryoprotectants[tiab] OR cryoprotection[tiab] OR vitrification[tiab] OR "post-thaw"[tiab] OR "post thaw"[tiab] OR "freeze-thaw"[tiab] OR "DMSO-free"[tiab] OR "DMSO free"[tiab] OR "reduced DMSO"[tiab] OR "low DMSO"[tiab] OR (("stem cell"[tiab] OR "stem cells"[tiab] OR "cell product"[tiab] OR "cell products"[tiab] OR "CAR-T"[tiab] OR "mesenchymal stromal cell"[tiab] OR "mesenchymal stromal cells"[tiab] OR "hematopoietic progenitor"[tiab] OR "haematopoietic progenitor"[tiab]) AND (freezing[tiab] OR thawing[tiab] OR "cell washing"[tiab] OR "DMSO removal"[tiab] OR "residual DMSO"[tiab]))) AND (human*[tiab] OR patient*[tiab] OR clinical[tiab] OR biomedical[tiab] OR mammalian[tiab] OR therapeutic[tiab] OR transplant*[tiab] OR transfusion*[tiab] OR "cell therapy"[tiab] OR "cell therapies"[tiab] OR "cell product"[tiab] OR "cell products"[tiab] OR "stem cell"[tiab] OR "stem cells"[tiab] OR hematopoietic[tiab] OR haematopoietic[tiab] OR progenitor[tiab] OR mesenchymal[tiab] OR "CAR-T"[tiab] OR "CAR T"[tiab] OR "T cell"[tiab] OR "T cells"[tiab] OR "NK cell"[tiab] OR "NK cells"[tiab] OR immune[tiab] OR platelet*[tiab] OR blood[tiab] OR erythrocyte*[tiab] OR leukocyte*[tiab] OR lymphocyte*[tiab] OR mononuclear[tiab] OR PBMC[tiab] OR "bone marrow"[tiab] OR iPSC[tiab] OR iPSCs[tiab] OR cardiomyocyte*[tiab] OR neuron*[tiab] OR nerve[tiab] OR "neural tissue"[tiab] OR "tissue construct"[tiab] OR "tissue constructs"[tiab] OR organoid*[tiab] OR graft*[tiab] OR "animal cell"[tiab] OR "animal cells"[tiab] OR "animal tissue"[tiab] OR "animal tissues"[tiab])) AND ((("Dimethyl Sulfoxide"[Majr] OR "dimethyl sulfoxide"[ti] OR "dimethyl sulphoxide"[ti] OR dimethylsulfoxide[ti] OR dimethylsulphoxide[ti] OR DMSO[ti] OR Me2SO[ti] OR "effect of DMSO"[tiab:~0] OR "effects of DMSO"[tiab:~0] OR "DMSO-induced"[tiab] OR "DMSO-mediated"[tiab] OR "DMSO exposure"[tiab] OR "DMSO treatment"[tiab] OR "DMSO concentration"[tiab] OR "DMSO concentrations"[tiab] OR "effects of dimethyl sulfoxide"[tiab:~0] OR "effect of dimethyl sulfoxide"[tiab:~0] OR "dimethyl sulfoxide-induced"[tiab] OR "dimethyl sulfoxide exposure"[tiab] OR "dimethyl sulfoxide treatment"[tiab]) OR ("DMSO-containing"[tiab] OR "DMSO containing"[tiab] OR "DMSO-based"[tiab] OR "DMSO based"[tiab] OR "DMSO formulation"[tiab] OR "DMSO formulations"[tiab:~0] OR "absorption enhancer"[tiab] OR "carrier containing DMSO"[tiab:~0])) OR (("dimethyl sulfoxide"[tiab] OR "dimethyl sulphoxide"[tiab] OR dimethylsulfoxide[tiab] OR dimethylsulphoxide[tiab] OR DMSO[tiab] OR Me2SO[tiab]) AND (cryoprotectant[tiab] OR cryoprotectants[tiab] OR "cryoprotective agent"[tiab] OR "cryoprotective agents"[tiab] OR "residual DMSO"[tiab] OR "DMSO removal"[tiab] OR "reduced	1,410

Database	Search Step	Direct-search format	Records
		DMSO"[tiab] OR "low DMSO"[tiab] OR "DMSO-free"[tiab] OR "DMSO free"[tiab])))) AND 1960/01/01:2026/10/12[dp]	
PubMed	S4. A AND F AND B2	(("Dimethyl Sulfoxide"[Mesh] OR "dimethyl sulfoxide"[tiab] OR "dimethyl sulphoxide"[tiab] OR dimethylsulfoxide[tiab] OR dimethylsulphoxide[tiab] OR DMSO[tiab] OR Me2SO[tiab]) AND (("Dimethyl Sulfoxide"[Majr] OR "dimethyl sulfoxide"[ti] OR "dimethyl sulphoxide"[ti] OR dimethylsulfoxide[ti] OR dimethylsulphoxide[ti] OR DMSO[ti] OR Me2SO[ti] OR "effect of DMSO"[tiab:~0] OR "effects of DMSO"[tiab:~0] OR "DMSO-induced"[tiab] OR "DMSO-mediated"[tiab] OR "DMSO exposure"[tiab] OR "DMSO treatment"[tiab] OR "DMSO concentration"[tiab] OR "DMSO concentrations"[tiab] OR "effects of dimethyl sulfoxide"[tiab:~0] OR "effect of dimethyl sulfoxide"[tiab:~0] OR "dimethyl sulfoxide-induced"[tiab] OR "dimethyl sulfoxide exposure"[tiab] OR "dimethyl sulfoxide treatment"[tiab]) OR ("DMSO-containing"[tiab] OR "DMSO containing"[tiab] OR "DMSO-based"[tiab] OR "DMSO based"[tiab] OR "DMSO formulation"[tiab] OR "DMSO formulations"[tiab:~0] OR "absorption enhancer"[tiab] OR "carrier containing DMSO"[tiab:~0])) AND ((osteoarthritis[tiab] OR arthritis[tiab] OR "systemic sclerosis"[tiab] OR "digital ulcer"[tiab] OR "digital ulcers"[tiab] OR musculoskeletal[tiab] OR "soft tissue injury"[tiab] OR "soft tissue injuries"[tiab] OR "traumatic brain injury"[tiab] OR "brain injury"[tiab] OR "spinal cord injury"[tiab] OR "intracranial pressure"[tiab] OR "cerebral edema"[tiab] OR "cerebral oedema"[tiab] OR "cerebral ischemia"[tiab] OR "cerebral ischaemia"[tiab] OR "myocardial ischemia"[tiab] OR "myocardial ischaemia"[tiab] OR "myocardial infarction"[tiab] OR "ischemia reperfusion"[tiab] OR "ischaemia reperfusion"[tiab] OR extravasation[tiab] OR "permeation enhancer"[tiab] OR "drug delivery"[tiab] OR transdermal[tiab] OR topical[tiab] OR ophthalmic[tiab] OR ocular[tiab] OR tinnitus[tiab] OR otic[tiab] OR onychomycosis[tiab] OR oncology[tiab] OR cancer[tiab] OR neoplasm[tiab] OR hematoxylin[tiab] OR haematoxylin[tiab] OR haematoxylon[tiab]) OR ("spinal cord trauma"[tiab] OR "myocardial necrosis"[tiab] OR "absorption enhancer"[tiab] OR "penetration enhancer"[tiab] OR "skin penetration"[tiab] OR "topical vehicle"[tiab]))) AND 1960/01/01:2026/10/12[dp]	770

Database	Search Step	Direct-search format	Records
PubMed	S5. A AND F AND B4	(("Dimethyl Sulfoxide"[Mesh] OR "dimethyl sulfoxide"[tiab] OR "dimethyl sulphoxide"[tiab] OR dimethylsulfoxide[tiab] OR dimethylsulphoxide[tiab] OR DMSO[tiab] OR Me2SO[tiab]) AND (("Dimethyl Sulfoxide"[Majr] OR "dimethyl sulfoxide"[ti] OR "dimethyl sulphoxide"[ti] OR dimethylsulfoxide[ti] OR dimethylsulphoxide[ti] OR DMSO[ti] OR Me2SO[ti] OR "effect of DMSO"[tiab:~0] OR "effects of DMSO"[tiab:~0] OR "DMSO-induced"[tiab] OR "DMSO-mediated"[tiab] OR "DMSO exposure"[tiab] OR "DMSO treatment"[tiab] OR "DMSO concentration"[tiab] OR "DMSO concentrations"[tiab] OR "effects of dimethyl sulfoxide"[tiab:~0] OR "effect of dimethyl sulfoxide"[tiab:~0] OR "dimethyl sulfoxide-induced"[tiab] OR "dimethyl sulfoxide exposure"[tiab] OR "dimethyl sulfoxide treatment"[tiab]) OR ("DMSO-containing"[tiab] OR "DMSO containing"[tiab] OR "DMSO-based"[tiab] OR "DMSO based"[tiab] OR "DMSO formulation"[tiab] OR "DMSO formulations"[tiab:~0] OR "absorption enhancer"[tiab] OR "carrier containing DMSO"[tiab:~0])) AND ((toxicity[tiab] OR toxic[tiab] OR safety[tiab] OR "adverse event"[tiab] OR "adverse events"[tiab] OR "adverse effect"[tiab] OR "adverse effects"[tiab] OR tolerability[tiab] OR cytotoxicity[tiab] OR hemolysis[tiab] OR haemolysis[tiab] OR irritation[tiab] OR "infusion reaction"[tiab] OR "infusion reactions"[tiab] OR "infusion-related"[tiab] OR "dose-dependent"[tiab] OR "concentration-dependent"[tiab]) OR (nephrotoxicity[tiab] OR nephrotoxic[tiab] OR hemoglobinemia[tiab] OR haemoglobinaemia[tiab] OR hemoglobinuria[tiab] OR haemoglobinuria[tiab] OR hyperosmolarity[tiab] OR hyperosmolality[tiab] OR "renal toxicity"[tiab] OR "renal safety"[tiab] OR "cardiac toxicity"[tiab] OR arrhythmia[tiab] OR hypotension[tiab] OR bradycardia[tiab] OR "neurological event"[tiab] OR "neurologic event"[tiab]))) AND 1960/01/01:2026/10/12[dp]	1,197

Database	Search Step	Direct-search format	Records
PubMed	S6. A AND F AND B5	(("Dimethyl Sulfoxide"[Mesh] OR "dimethyl sulfoxide"[tiab] OR "dimethyl sulphoxide"[tiab] OR dimethylsulfoxide[tiab] OR dimethylsulphoxide[tiab] OR DMSO[tiab] OR Me2SO[tiab]) AND (("Dimethyl Sulfoxide"[Majr] OR "dimethyl sulfoxide"[ti] OR "dimethyl sulphoxide"[ti] OR dimethylsulfoxide[ti] OR dimethylsulphoxide[ti] OR DMSO[ti] OR Me2SO[ti] OR "effect of DMSO"[tiab:~0] OR "effects of DMSO"[tiab:~0] OR "DMSO-induced"[tiab] OR "DMSO-mediated"[tiab] OR "DMSO exposure"[tiab] OR "DMSO treatment"[tiab] OR "DMSO concentration"[tiab] OR "DMSO concentrations"[tiab] OR "effects of dimethyl sulfoxide"[tiab:~0] OR "effect of dimethyl sulfoxide"[tiab:~0] OR "dimethyl sulfoxide-induced"[tiab] OR "dimethyl sulfoxide exposure"[tiab] OR "dimethyl sulfoxide treatment"[tiab]) OR ("DMSO-containing"[tiab] OR "DMSO containing"[tiab] OR "DMSO-based"[tiab] OR "DMSO based"[tiab] OR "DMSO formulation"[tiab] OR "DMSO formulations"[tiab:~0] OR "absorption enhancer"[tiab] OR "carrier containing DMSO"[tiab:~0])) AND ("Cell Membrane"[Mesh] OR "High-Throughput Screening Assays"[Mesh] OR "membrane permeability"[tiab] OR "membrane fluidity"[tiab] OR "membrane integrity"[tiab] OR "lipid bilayer"[tiab] OR "lipid bilayers"[tiab] OR "protein stability"[tiab] OR "protein conformation"[tiab] OR "protein folding"[tiab] OR "protein aggregation"[tiab] OR "protein denaturation"[tiab] OR "ligand binding"[tiab] OR "binding kinetics"[tiab] OR "solvent effect"[tiab] OR "solvent effects"[tiab] OR "vehicle effect"[tiab] OR "vehicle effects"[tiab] OR "assay interference"[tiab] OR "assay bias"[tiab] OR "high-throughput screening"[tiab] OR "high throughput screening"[tiab] OR "fragment-based screening"[tiab] OR "drug screening"[tiab] OR "oxidative stress"[tiab] OR "cellular stress"[tiab] OR "gene expression"[tiab] OR epigenetic[tiab] OR epigenetics[tiab] OR inflammation[tiab] OR inflammatory[tiab] OR cytokine[tiab] OR cytokines[tiab] OR "cell differentiation"[tiab] OR "cell viability"[tiab] OR mitochondrial[tiab] OR "alpha-synuclein"[tiab] OR "amyloid beta"[tiab])) AND 1960/01/01:2026/10/12[dp]	1,433

PubMed	Final fixed union	("Dimethyl Sulfoxide"[Mesh] OR "dimethyl sulfoxide"[tiab] OR "dimethyl sulphoxide"[tiab] OR dimethylsulfoxide[tiab] OR dimethylsulphoxide[tiab] OR DMSO[tiab] OR Me2SO[tiab]) AND (("Cystitis, Interstitial"[Mesh] OR "interstitial cystitis"[tiab] OR "bladder pain syndrome"[tiab] OR "painful bladder syndrome"[tiab] OR "IC/BPS"[tiab] OR "IC/PBS"[tiab] OR "Hunner lesion"[tiab] OR "Hunner lesions"[tiab] OR "Hunner disease"[tiab:~0] OR intravesical[tiab] OR "bladder instillation"[tiab] OR "bladder instillations"[tiab] OR "KRP-116D"[tiab]) OR ((("Cryopreservation"[Mesh] OR cryopreservation[tiab] OR cryopreserved[tiab] OR cryoprotectant[tiab] OR cryoprotectants[tiab] OR cryoprotection[tiab] OR vitrification[tiab] OR "post-thaw"[tiab] OR "post thaw"[tiab] OR "freeze-thaw"[tiab] OR "DMSO-free"[tiab] OR "DMSO free"[tiab] OR "reduced DMSO"[tiab] OR "low DMSO"[tiab] OR (("stem cell"[tiab] OR "stem cells"[tiab] OR "cell product"[tiab] OR "cell products"[tiab] OR "CAR-T"[tiab] OR "mesenchymal stromal cell"[tiab] OR "mesenchymal stromal cells"[tiab] OR "hematopoietic progenitor"[tiab] OR "haematopoietic progenitor"[tiab]) AND (freezing[tiab] OR thawing[tiab] OR "cell washing"[tiab] OR "DMSO removal"[tiab] OR "residual DMSO"[tiab]))) AND (human*[tiab] OR patient*[tiab] OR clinical[tiab] OR biomedical[tiab] OR mammalian[tiab] OR therapeutic[tiab] OR transplant*[tiab] OR transfusion*[tiab] OR "cell therapy"[tiab] OR "cell therapies"[tiab] OR "cell product"[tiab] OR "cell products"[tiab] OR "stem cell"[tiab] OR "stem cells"[tiab] OR hematopoietic[tiab] OR haematopoietic[tiab] OR progenitor[tiab] OR mesenchymal[tiab] OR "CAR-T"[tiab] OR "CAR T"[tiab] OR "T cell"[tiab] OR "T cells"[tiab] OR "NK cell"[tiab] OR "NK cells"[tiab] OR immune[tiab] OR platelet*[tiab] OR blood[tiab] OR erythrocyte*[tiab] OR leukocyte*[tiab] OR lymphocyte*[tiab] OR mononuclear[tiab] OR PBMC[tiab] OR "bone marrow"[tiab] OR iPSC[tiab] OR iPSCs[tiab] OR cardiomyocyte*[tiab] OR neuron*[tiab] OR nerve[tiab] OR "neural tissue"[tiab] OR "tissue construct"[tiab] OR "tissue constructs"[tiab] OR organoid*[tiab] OR graft*[tiab] OR "animal cell"[tiab] OR "animal cells"[tiab] OR "animal tissue"[tiab] OR "animal tissues"[tiab])) AND ((("Dimethyl Sulfoxide"[Majr] OR "dimethyl sulfoxide"[ti] OR "dimethyl sulphoxide"[ti] OR dimethylsulfoxide[ti] OR dimethylsulphoxide[ti] OR DMSO[ti] OR Me2SO[ti] OR "effect of DMSO"[tiab:~0] OR "effects of DMSO"[tiab:~0] OR "DMSO-induced"[tiab] OR "DMSO-mediated"[tiab] OR "DMSO exposure"[tiab] OR "DMSO treatment"[tiab] OR "DMSO concentration"[tiab] OR "DMSO concentrations"[tiab] OR "effects of dimethyl sulfoxide"[tiab:~0] OR "effect of dimethyl sulfoxide"[tiab:~0] OR "dimethyl sulfoxide-induced"[tiab] OR "dimethyl sulfoxide exposure"[tiab] OR "dimethyl sulfoxide treatment"[tiab]) OR ("DMSO-containing"[tiab] OR "DMSO containing"[tiab] OR "DMSO-based"[tiab] OR "DMSO based"[tiab] OR "DMSO formulation"[tiab] OR "DMSO formulations"[tiab:~0] OR "absorption enhancer"[tiab] OR "carrier containing DMSO"[tiab:~0])) OR (("dimethyl sulfoxide"[tiab] OR "dimethyl sulphoxide"[tiab] OR dimethylsulfoxide[tiab] OR dimethylsulphoxide[tiab] OR	4,052

DMSO[tiab] OR Me2SO[tiab]) AND (cryoprotectant[tiab] OR cryoprotectants[tiab] OR "cryoprotective agent"[tiab] OR "cryoprotective agents"[tiab] OR "residual DMSO"[tiab] OR "DMSO removal"[tiab] OR "reduced DMSO"[tiab] OR "low DMSO"[tiab] OR "DMSO-free"[tiab] OR "DMSO free"[tiab])))) OR ((("Dimethyl Sulfoxide"[Majr] OR "dimethyl sulfoxide"[ti] OR "dimethyl sulphoxide"[ti] OR dimethylsulfoxide[ti] OR dimethylsulphoxide[ti] OR DMSO[ti] OR Me2SO[ti] OR "effect of DMSO"[tiab:~0] OR "effects of DMSO"[tiab:~0] OR "DMSO-induced"[tiab] OR "DMSO-mediated"[tiab] OR "DMSO exposure"[tiab] OR "DMSO treatment"[tiab] OR "DMSO concentration"[tiab] OR "DMSO concentrations"[tiab] OR "effects of dimethyl sulfoxide"[tiab:~0] OR "effect of dimethyl sulfoxide"[tiab:~0] OR "dimethyl sulfoxide-induced"[tiab] OR "dimethyl sulfoxide exposure"[tiab] OR "dimethyl sulfoxide treatment"[tiab]) OR ("DMSO-containing"[tiab] OR "DMSO containing"[tiab] OR "DMSO-based"[tiab] OR "DMSO based"[tiab] OR "DMSO formulation"[tiab] OR "DMSO formulations"[tiab:~0] OR "absorption enhancer"[tiab] OR "carrier containing DMSO"[tiab:~0])) AND (((osteoarthritis[tiab] OR arthritis[tiab] OR "systemic sclerosis"[tiab] OR "digital ulcer"[tiab] OR "digital ulcers"[tiab] OR musculoskeletal[tiab] OR "soft tissue injury"[tiab] OR "soft tissue injuries"[tiab] OR "traumatic brain injury"[tiab] OR "brain injury"[tiab] OR "spinal cord injury"[tiab] OR "intracranial pressure"[tiab] OR "cerebral edema"[tiab] OR "cerebral oedema"[tiab] OR "cerebral ischemia"[tiab] OR "cerebral ischaemia"[tiab] OR "myocardial ischemia"[tiab] OR "myocardial ischaemia"[tiab] OR "myocardial infarction"[tiab] OR "ischemia reperfusion"[tiab] OR "ischaemia reperfusion"[tiab] OR extravasation[tiab] OR "permeation enhancer"[tiab] OR "drug delivery"[tiab] OR transdermal[tiab] OR topical[tiab] OR ophthalmic[tiab] OR ocular[tiab] OR tinnitus[tiab] OR otic[tiab] OR onychomycosis[tiab] OR oncology[tiab] OR cancer[tiab] OR neoplasm[tiab] OR hematoxylin[tiab] OR haematoxylin[tiab] OR haematoxylon[tiab]) OR ("spinal cord trauma"[tiab] OR "myocardial necrosis"[tiab] OR "absorption enhancer"[tiab] OR "penetration enhancer"[tiab] OR "skin penetration"[tiab] OR "topical vehicle"[tiab])) OR ((toxicity[tiab] OR toxic[tiab] OR safety[tiab] OR "adverse event"[tiab] OR "adverse events"[tiab] OR "adverse effect"[tiab] OR "adverse effects"[tiab] OR tolerability[tiab] OR cytotoxicity[tiab] OR hemolysis[tiab] OR haemolysis[tiab] OR irritation[tiab] OR "infusion reaction"[tiab] OR "infusion reactions"[tiab] OR "infusion-related"[tiab] OR "dose-dependent"[tiab] OR "concentration-dependent"[tiab]) OR (nephrotoxicity[tiab] OR nephrotoxic[tiab] OR hemoglobinemia[tiab] OR haemoglobinaemia[tiab] OR hemoglobinuria[tiab] OR haemoglobinuria[tiab] OR hyperosmolarity[tiab] OR hyperosmolality[tiab] OR "renal toxicity"[tiab] OR "renal safety"[tiab] OR "cardiac toxicity"[tiab] OR arrhythmia[tiab] OR hypotension[tiab] OR bradycardia[tiab] OR "neurological event"[tiab] OR "neurologic event"[tiab])) OR ("Cell Membrane"[Mesh] OR "High-Throughput

Database	Search Step	Direct-search format	Records
		Screening Assays"[Mesh] OR "membrane permeability"[tiab] OR "membrane fluidity"[tiab] OR "membrane integrity"[tiab] OR "lipid bilayer"[tiab] OR "lipid bilayers"[tiab] OR "protein stability"[tiab] OR "protein conformation"[tiab] OR "protein folding"[tiab] OR "protein aggregation"[tiab] OR "protein denaturation"[tiab] OR "ligand binding"[tiab] OR "binding kinetics"[tiab] OR "solvent effect"[tiab] OR "solvent effects"[tiab] OR "vehicle effect"[tiab] OR "vehicle effects"[tiab] OR "assay interference"[tiab] OR "assay bias"[tiab] OR "high-throughput screening"[tiab] OR "high throughput screening"[tiab] OR "fragment-based screening"[tiab] OR "drug screening"[tiab] OR "oxidative stress"[tiab] OR "cellular stress"[tiab] OR "gene expression"[tiab] OR epigenetic[tiab] OR epigenetics[tiab] OR inflammation[tiab] OR inflammatory[tiab] OR cytokine[tiab] OR cytokines[tiab] OR "cell differentiation"[tiab] OR "cell viability"[tiab] OR mitochondrial[tiab] OR "alpha-synuclein"[tiab] OR "amyloid beta"[tiab])))) AND 1960/01/01:2026/10/12[dp]	
Web of Science Core Collection	S1. Core A	TS=("dimethyl sulfoxide" OR "dimethyl sulphoxide" OR dimethylsulfoxide OR dimethylsulphoxide OR DMSO OR Me2SO); Timespan = 1960–2026	62,036
Web of Science Core Collection	S2. A AND B1	TS=("dimethyl sulfoxide" OR "dimethyl sulphoxide" OR dimethylsulfoxide OR dimethylsulphoxide OR DMSO OR Me2SO) AND TS=("interstitial cystitis" OR "bladder pain syndrome" OR "painful bladder syndrome" OR "IC/BPS" OR "IC/PBS" OR "Hunner lesion*" OR "Hunner ulcer*" OR "Hunner disease" OR intravesical OR "bladder instillation*" OR "KRP-116D"); Timespan = 1960–2026	194
Web of Science Core Collection	S3. A AND B3-biomedical AND cryopreservation-focus	TS=("dimethyl sulfoxide" OR "dimethyl sulphoxide" OR dimethylsulfoxide OR dimethylsulphoxide OR DMSO OR Me2SO) AND TS=(human* OR patient* OR clinical OR biomedical OR mammalian OR therapeutic OR transplant* OR transfusion* OR "cell therap*" OR "cell product*" OR "stem cell*" OR hematopoietic OR haematopoietic OR progenitor OR mesenchymal OR "CAR-T" OR "CAR T" OR "T cell*" OR "NK cell*" OR platelet* OR erythrocyte* OR leukocyte* OR lymphocyte* OR mononuclear OR PBMC OR "bone marrow" OR iPSC* OR cardiomyocyte* OR neuron* OR "neural tissue" OR "tissue construct*" OR organoid* OR graft* OR "animal cell*" OR "animal tissue*") AND TS=(cryopreserv* OR cryoprotect* OR vitrification OR "post-thaw" OR "post thaw" OR "freeze-thaw" OR "DMSO-free" OR "DMSO free" OR "reduced DMSO" OR "low DMSO" OR "DMSO removal" OR "residual DMSO") AND (TI=("dimethyl sulfoxide" OR "dimethyl sulphoxide" OR dimethylsulfoxide OR dimethylsulphoxide OR DMSO OR Me2SO) OR AB=(("dimethyl sulfoxide" OR "dimethyl sulphoxide" OR dimethylsulfoxide OR dimethylsulphoxide OR DMSO OR Me2SO) NEAR/5 (cryopreserv* OR cryoprotect* OR vitrification OR freez* OR thaw* OR residual OR removal OR reduced))); Timespan = 1960–2026	1,494

Database	Search Step	Direct-search format	Records
Web of Science Core Collection	S4. A AND F AND B2	TS=("dimethyl sulfoxide" OR "dimethyl sulphoxide" OR dimethylsulfoxide OR dimethylsulphoxide OR DMSO OR Me2SO) AND ((TI=("dimethyl sulfoxide" OR "dimethyl sulphoxide" OR dimethylsulfoxide OR dimethylsulphoxide OR DMSO OR Me2SO) AND TS=(osteoarthritis OR arthritis OR "systemic sclerosis" OR "digital ulcer*" OR musculoskeletal OR "soft tissue injur*" OR "traumatic brain injury" OR "brain injury" OR "spinal cord injury" OR "spinal cord trauma" OR "intracranial pressure" OR "cerebral edema" OR "cerebral oedema" OR "cerebral ischemia" OR "cerebral ischaemia" OR "myocardial ischemia" OR "myocardial ischaemia" OR "myocardial infarction" OR "myocardial necrosis" OR "ischemia reperfusion" OR "ischaemia reperfusion" OR extravasation OR "permeation enhancer*" OR "absorption enhancer*" OR "penetration enhancer*" OR "skin penetration" OR "drug delivery" OR transdermal OR topical OR "topical vehicle" OR ophthalmic OR ocular OR tinnitus OR otic OR onychomycosis OR oncology OR cancer OR neoplasm* OR hematoxylin OR haematoxylin OR haematoxylon)) OR AB=(("dimethyl sulfoxide" OR "dimethyl sulphoxide" OR dimethylsulfoxide OR dimethylsulphoxide OR DMSO OR Me2SO) NEAR/5 (osteoarthritis OR arthritis OR "systemic sclerosis" OR "digital ulcer*" OR musculoskeletal OR "soft tissue injur*" OR "traumatic brain injury" OR "brain injury" OR "spinal cord injury" OR "spinal cord trauma" OR "intracranial pressure" OR "cerebral edema" OR "cerebral oedema" OR "cerebral ischemia" OR "cerebral ischaemia" OR "myocardial ischemia" OR "myocardial ischaemia" OR "myocardial infarction" OR "myocardial necrosis" OR "ischemia reperfusion" OR "ischaemia reperfusion" OR extravasation OR "permeation enhancer*" OR "absorption enhancer*" OR "penetration enhancer*" OR "skin penetration" OR "drug delivery" OR transdermal OR topical OR "topical vehicle" OR ophthalmic OR ocular OR tinnitus OR otic OR onychomycosis OR oncology OR cancer OR neoplasm* OR hematoxylin OR haematoxylin OR haematoxylon))); Timespan = 1960–2026	587

Database	Search Step	Direct-search format	Records
Web of Science Core Collection	S5. A AND F AND B4	TS=("dimethyl sulfoxide" OR "dimethyl sulphoxide" OR dimethylsulfoxide OR dimethylsulphoxide OR DMSO OR Me2SO) AND ((TI=("dimethyl sulfoxide" OR "dimethyl sulphoxide" OR dimethylsulfoxide OR dimethylsulphoxide OR DMSO OR Me2SO) AND TS=(toxicity OR toxic* OR safety OR "adverse event*" OR "adverse effect*" OR tolerability OR cytotoxic* OR hemolysis OR haemolysis OR irritation OR "infusion reaction*" OR "infusion-related" OR "dose-dependent" OR "concentration-dependent" OR nephrotoxic* OR hemoglobinemia OR haemoglobinaemia OR hemoglobinuria OR haemoglobinuria OR hyperosmolarity OR hyperosmolality OR "renal toxicity" OR "renal safety" OR "cardiac toxicity" OR arrhythmia OR hypotension OR bradycardia OR "neurological event*" OR "neurologic event*")) OR AB=(("dimethyl sulfoxide" OR "dimethyl sulphoxide" OR dimethylsulfoxide OR dimethylsulphoxide OR DMSO OR Me2SO) NEAR/5 (toxicity OR toxic* OR safety OR "adverse event*" OR "adverse effect*" OR tolerability OR cytotoxic* OR hemolysis OR haemolysis OR irritation OR "infusion reaction*" OR "infusion-related" OR "dose-dependent" OR "concentration-dependent" OR nephrotoxic* OR hemoglobinemia OR haemoglobinaemia OR hemoglobinuria OR haemoglobinuria OR hyperosmolarity OR hyperosmolality OR "renal toxicity" OR "renal safety" OR "cardiac toxicity" OR arrhythmia OR hypotension OR bradycardia OR "neurological event*" OR "neurologic event*"))); Timespan = 1960–2026	1,401

Database	Search Step	Direct-search format	Records
Web of Science Core Collection	S6. A AND F AND B5	TS=("dimethyl sulfoxide" OR "dimethyl sulphoxide" OR dimethylsulfoxide OR dimethylsulphoxide OR DMSO OR Me2SO) AND ((TI=("dimethyl sulfoxide" OR "dimethyl sulphoxide" OR dimethylsulfoxide OR dimethylsulphoxide OR DMSO OR Me2SO) AND TS=("cell membrane" OR "membrane permeability" OR "membrane fluidity" OR "membrane integrity" OR "lipid bilayer*" OR "protein stability" OR "protein conformation" OR "protein folding" OR "protein aggregation" OR "protein denaturation" OR "ligand binding" OR "binding kinetics" OR "solvent effect*" OR "vehicle effect*" OR "assay interference" OR "assay bias" OR "high-throughput screening" OR "high throughput screening" OR "fragment-based screening" OR "drug screening" OR "oxidative stress" OR "cellular stress" OR "gene expression" OR epigenetic* OR inflammation OR inflammatory OR cytokine* OR "cell differentiation" OR "cell viability" OR mitochondrial OR "alpha-synuclein" OR "amyloid beta")) OR AB=(("dimethyl sulfoxide" OR "dimethyl sulphoxide" OR dimethylsulfoxide OR dimethylsulphoxide OR DMSO OR Me2SO) NEAR/5 ("cell membrane" OR "membrane permeability" OR "membrane fluidity" OR "membrane integrity" OR "lipid bilayer*" OR "protein stability" OR "protein conformation" OR "protein folding" OR "protein aggregation" OR "protein denaturation" OR "ligand binding" OR "binding kinetics" OR "solvent effect*" OR "vehicle effect*" OR "assay interference" OR "assay bias" OR "high-throughput screening" OR "high throughput screening" OR "fragment-based screening" OR "drug screening" OR "oxidative stress" OR "cellular stress" OR "gene expression" OR epigenetic* OR inflammation OR inflammatory OR cytokine* OR "cell differentiation" OR "cell viability" OR mitochondrial OR "alpha-synuclein" OR "amyloid beta"))); Timespan = 1960–2026	1,207
Web of Science Core Collection	Final fixed union	#2 OR #11 OR #10 OR #12 OR #13 (validated saved-set union of S2–S6; all component sets used Web of Science Core Collection and Timespan = 1960–2026)	3,992
ClinicalTrials.gov	S1. Core A	AREA[InterventionSearch]("dimethyl sulfoxide" OR "dimethyl sulphoxide" OR dimethylsulfoxide OR dimethylsulphoxide OR DMSO OR Me2SO)	138
ClinicalTrials.gov	S2. A AND B1	AREA[InterventionSearch]("dimethyl sulfoxide" OR "dimethyl sulphoxide" OR dimethylsulfoxide OR dimethylsulphoxide OR DMSO OR Me2SO) AND AREA[BasicSearch]("interstitial cystitis" OR "bladder pain syndrome" OR "painful bladder syndrome" OR "IC/BPS" OR "IC-PBS" OR "Hunner lesion" OR "Hunner lesions" OR "Hunner disease" OR intravesical OR "intravesical instillation" OR "intravesical instillations" OR "bladder instillation" OR "bladder instillations" OR KRP-116D)	9

Database	Search Step	Direct-search format	Records
ClinicalTrials.gov	S3. A AND B3-biomedical AND cryopreservation-focus	AREA[InterventionSearch]("dimethyl sulfoxide" OR "dimethyl sulphoxide" OR dimethylsulfoxide OR dimethylsulphoxide OR DMSO OR Me2SO) AND AREA[BasicSearch]("cell product" OR "cell products" OR "hematopoietic stem cell" OR "hematopoietic stem cells" OR "haematopoietic stem cell" OR "haematopoietic stem cells" OR "hematopoietic progenitor cell" OR "hematopoietic progenitor cells" OR "haematopoietic progenitor cell" OR "haematopoietic progenitor cells" OR PBSC OR graft OR grafts OR "CAR-T" OR "CAR T" OR "T cell" OR "T cells" OR "NK cell" OR "NK cells" OR iPSC OR "induced pluripotent stem cell" OR "induced pluripotent stem cells" OR "mesenchymal stem cell" OR "mesenchymal stem cells" OR "mesenchymal stromal cell" OR "mesenchymal stromal cells" OR cardiomyocyte OR cardiomyocytes OR transplant OR transplantation OR transfusion) AND AREA[BasicSearch](cryopreservation OR cryopreserved OR cryoprotectant OR cryoprotectants OR cryoprotection OR vitrification OR "post-thaw" OR "post thaw" OR "DMSO-free" OR "DMSO free" OR "reduced DMSO" OR "low DMSO" OR "DMSO removal" OR "residual DMSO" OR "DMSO concentration" OR "DMSO concentrations")	16
ClinicalTrials.gov	S4. A AND F AND B2	AREA[InterventionSearch]("dimethyl sulfoxide" OR "dimethyl sulphoxide" OR dimethylsulfoxide OR dimethylsulphoxide OR DMSO OR Me2SO) AND AREA[BasicSearch]("DMSO treatment" OR "DMSO exposure" OR "DMSO concentration" OR "DMSO concentrations" OR "DMSO-containing" OR "DMSO containing" OR "DMSO-based" OR "DMSO based" OR "dimethyl sulfoxide treatment" OR "dimethyl sulfoxide exposure" OR "dimethyl sulfoxide concentration" OR "dimethyl sulphoxide treatment" OR "dimethyl sulphoxide exposure") AND AREA[BasicSearch](pain OR analgesia OR musculoskeletal OR arthritis OR osteoarthritis OR gonarthrosis OR "rheumatoid arthritis" OR "systemic sclerosis" OR "digital ulcer" OR "digital ulcers" OR "soft tissue" OR "soft-tissue" OR "traumatic injury" OR sprain OR sprains OR strain OR strains OR contusion OR contusions OR "cerebral edema" OR "cerebral oedema" OR "brain injury" OR "head trauma" OR "traumatic brain injury" OR "spinal cord injury" OR "myocardial infarction" OR "myocardial ischemia" OR "myocardial ischaemia" OR stroke OR "ischemia reperfusion" OR "ischaemia reperfusion" OR extravasation OR "drug delivery" OR transdermal OR topical OR ophthalmic OR ocular OR tinnitus OR otic OR onychomycosis OR oncology OR cancer OR neoplasm OR hematoxylin OR haematoxylin OR haematoxylon)	4

Database	Search Step	Direct-search format	Records
ClinicalTrials.gov	S5. A AND F AND B4	AREA[InterventionSearch]("dimethyl sulfoxide" OR "dimethyl sulphoxide" OR dimethylsulfoxide OR dimethylsulphoxide OR DMSO OR Me2SO) AND AREA[BasicSearch]("DMSO treatment" OR "DMSO exposure" OR "DMSO concentration" OR "DMSO concentrations" OR "DMSO-containing" OR "DMSO containing" OR "DMSO-based" OR "DMSO based" OR "dimethyl sulfoxide treatment" OR "dimethyl sulfoxide exposure" OR "dimethyl sulfoxide concentration" OR "dimethyl sulphoxide treatment" OR "dimethyl sulphoxide exposure") AND AREA[BasicSearch](toxicity OR toxic OR safety OR "adverse event" OR "adverse events" OR "adverse effect" OR "adverse effects" OR tolerability OR intolerance OR irritation OR dermatitis OR erythema OR hemolysis OR haemolysis OR hemoglobinuria OR haemoglobinuria OR hyperosmolality OR osmolality OR electrolyte OR electrolytes OR nausea OR vomiting OR flushing OR hypoxia OR hypotension OR hypertension OR bradycardia OR tachycardia OR arrhythmia OR "infusion reaction" OR "infusion reactions" OR "infusion-related" OR renal OR kidney OR cardiac OR neurologic OR neurological)	3
ClinicalTrials.gov	S6. A AND F AND B5	AREA[InterventionSearch]("dimethyl sulfoxide" OR "dimethyl sulphoxide" OR dimethylsulfoxide OR dimethylsulphoxide OR DMSO OR Me2SO) AND AREA[BasicSearch]("DMSO treatment" OR "DMSO exposure" OR "DMSO concentration" OR "DMSO concentrations" OR "DMSO-containing" OR "DMSO containing" OR "DMSO-based" OR "DMSO based" OR "dimethyl sulfoxide treatment" OR "dimethyl sulfoxide exposure" OR "dimethyl sulfoxide concentration" OR "dimethyl sulphoxide treatment" OR "dimethyl sulphoxide exposure") AND AREA[BasicSearch](membrane OR membranes OR lipid OR lipids OR bilayer OR bilayers OR permeability OR permeation OR poration OR "pore formation" OR dehydration OR hydration OR protein OR proteins OR "protein interaction" OR "protein interactions" OR conformation OR stability OR "ligand binding" OR "binding kinetics" OR solubility OR precipitation OR aggregation OR "protein aggregation" OR inflammation OR inflammatory OR cytokine OR cytokines OR "oxidative stress" OR apoptosis OR differentiation OR "gene expression" OR epigenetic OR epigenetics OR "cell signaling" OR "cell signalling" OR "signal transduction" OR cytotoxicity OR viability OR metabolism OR metabolic OR enzyme OR enzymes OR receptor OR receptors OR "high-throughput screening" OR "high throughput screening" OR "assay interference" OR "vehicle effect" OR "solvent effect")	4

ClinicalTrials.gov	Final fixed union	AREA[InterventionSearch]("dimethyl sulfoxide" OR "dimethyl sulphoxide" OR dimethylsulfoxide OR dimethylsulphoxide OR DMSO OR Me2SO) AND (AREA[BasicSearch]("interstitial cystitis" OR "bladder pain syndrome" OR "painful bladder syndrome" OR "IC/BPS" OR "IC-PBS" OR "Hunner lesion" OR "Hunner lesions" OR "Hunner disease" OR intravesical OR "intravesical instillation" OR "intravesical instillations" OR "bladder instillation" OR "bladder instillations" OR KRP-116D) OR (AREA[BasicSearch]("cell product" OR "cell products" OR "hematopoietic stem cell" OR "hematopoietic stem cells" OR "haematopoietic stem cell" OR "haematopoietic stem cells" OR "hematopoietic progenitor cell" OR "hematopoietic progenitor cells" OR "haematopoietic progenitor cell" OR "haematopoietic progenitor cells" OR PBSC OR graft OR grafts OR "CAR-T" OR "CAR T" OR "T cell" OR "T cells" OR "NK cell" OR "NK cells" OR iPSC OR "induced pluripotent stem cell" OR "induced pluripotent stem cells" OR "mesenchymal stem cell" OR "mesenchymal stem cells" OR "mesenchymal stromal cell" OR "mesenchymal stromal cells" OR cardiomyocyte OR cardiomyocytes OR transplant OR transplantation OR transfusion) AND AREA[BasicSearch](cryopreservation OR cryopreserved OR cryoprotectant OR cryoprotectants OR cryoprotection OR vitrification OR "post-thaw" OR "post thaw" OR "DMSO-free" OR "DMSO free" OR "reduced DMSO" OR "low DMSO" OR "DMSO removal" OR "residual DMSO" OR "DMSO concentration" OR "DMSO concentrations")) OR (AREA[BasicSearch]("DMSO treatment" OR "DMSO exposure" OR "DMSO concentration" OR "DMSO concentrations" OR "DMSO-containing" OR "DMSO containing" OR "DMSO-based" OR "DMSO based" OR "dimethyl sulfoxide treatment" OR "dimethyl sulfoxide exposure" OR "dimethyl sulfoxide concentration" OR "dimethyl sulphoxide treatment" OR "dimethyl sulphoxide exposure") AND (AREA[BasicSearch](pain OR analgesia OR musculoskeletal OR arthritis OR osteoarthritis OR gonarthrosis OR "rheumatoid arthritis" OR "systemic sclerosis" OR "digital ulcer" OR "digital ulcers" OR "soft tissue" OR "soft-tissue" OR "traumatic injury" OR sprain OR sprains OR strain OR strains OR contusion OR contusions OR "cerebral edema" OR "cerebral oedema" OR "brain injury" OR "head trauma" OR "traumatic brain injury" OR "spinal cord injury" OR "myocardial infarction" OR "myocardial ischemia" OR "myocardial ischaemia" OR stroke OR "ischemia reperfusion" OR "ischaemia reperfusion" OR extravasation OR "drug delivery" OR transdermal OR topical OR ophthalmic OR ocular OR tinnitus OR otic OR onychomycosis OR oncology OR cancer OR neoplasm OR hematoxylin OR haematoxylin OR haematoxylon) OR AREA[BasicSearch](toxicity OR toxic OR safety OR "adverse event" OR "adverse events" OR "adverse effect" OR "adverse effects" OR tolerability OR intolerance OR irritation OR dermatitis OR erythema OR hemolysis OR haemolysis OR hemoglobinuria OR haemoglobinuria OR hyperosmolality OR osmolality OR electrolyte OR electrolytes OR nausea OR vomiting OR	27

Database	Search Step	Direct-search format	Records
		flushing OR hypoxia OR hypotension OR hypertension OR bradycardia OR tachycardia OR arrhythmia OR "infusion reaction" OR "infusion reactions" OR "infusion-related" OR renal OR kidney OR cardiac OR neurologic OR neurological) OR AREA[BasicSearch](membrane OR membranes OR lipid OR lipids OR bilayer OR bilayers OR permeability OR permeation OR poration OR "pore formation" OR dehydration OR hydration OR protein OR proteins OR "protein interaction" OR "protein interactions" OR conformation OR stability OR "ligand binding" OR "binding kinetics" OR solubility OR precipitation OR aggregation OR "protein aggregation" OR inflammation OR inflammatory OR cytokine OR cytokines OR "oxidative stress" OR apoptosis OR differentiation OR "gene expression" OR epigenetic OR epigenetics OR "cell signaling" OR "cell signalling" OR "signal transduction" OR cytotoxicity OR viability OR metabolism OR metabolic OR enzyme OR enzymes OR receptor OR receptors OR "high-throughput screening" OR "high throughput screening" OR "assay interference" OR "vehicle effect" OR "solvent effect"))))	

Note: Search blocks were applied across PubMed, Web of Science Core Collection, and ClinicalTrials.gov using platform-specific syntax and fields. PubMed and Web of Science Core Collection were searched on 12 October 2026, and ClinicalTrials.gov was searched on 13 October 2026. A = DMSO terms; B1 = IC/BPS and intravesical treatment; B2 = other clinical and historical applications; B3-biomedical = biomedical cryopreservation and cell-product handling; B4 = safety, toxicity, and tolerability; B5 = mechanisms and assay interference; and F = DMSO-centered focus terms. The B3 branch additionally required a cryopreservation-focus condition. In ClinicalTrials.gov, restriction to DMSO in the Intervention/treatment field served as the DMSO-centered focus, so F was not repeated in the Other terms field. B1S was used only for sensitivity searching and was not included in the final union. Database-specific field tags and syntax were adapted to each platform. Individual thematic searches overlap and should not be summed to obtain the number of unique records.
